# Sphingolipid Remodeling in Colorectal Cancer Reveals a Continuum-like Metabolic Organization

**DOI:** 10.3390/antiox15091136

**Published:** 2026-09-08

**Authors:** Adam R. Markowski, Karolina Stępniak, Piotr Zabielski, Hady Razak Hady, Aleksander Łukaszewicz, Paulina Głuszyńska, Patrycja Sadowska, Urszula Chlabicz, Agnieszka Błachnio-Zabielska

**Affiliations:** 1 Department of Hypertensiology, Gastroenterology and Internal Medicine, Medical University of Bialystok, 15-540 Bialystok, Poland; 2 Department of Hygiene, Epidemiology and Metabolic Disorders, Medical University of Bialystok, 15-222 Bialystok, Poland; karolina.stepniak@umb.edu.pl (K.S.); patrycja.sadowska@umb.edu.pl (P.S.); urszula.chlabicz@umb.edu.pl (U.C.); agnieszka.blachnio-zabielska@umb.edu.pl (A.B.-Z.); 3 Department of Medical Biology, Medical University of Bialystok, 15-222 Bialystok, Poland; piotr.zabielski@umb.edu.pl; 4 2nd Clinical Department of General, Gastroenterological, and Oncological Surgery, Medical University of Bialystok, 15-276 Bialystok, Poland; hady.razak-hady@umb.edu.pl (H.R.H.); alexander.luk6@gmail.com (A.Ł.); paulina.gluszynska@umb.edu.pl (P.G.)

**Keywords:** colorectal cancer, sphingolipids, ceramides, sphingosine-1-phosphate, lipidomics, cancer metabolism, biomarkers, oxidative stress, metabolic heterogeneity, systems biology

## Abstract

Colorectal cancer (CRC) exhibits substantial metabolic heterogeneity, but the organization of sphingolipid remodeling remains incompletely understood. In this exploratory single-center study, we integrated patient-matched tissue lipidomics, systemic oxidative stress profiling, and independent transcriptomic analyses. Tumor tissue, adjacent non-neoplastic mucosa, and preoperative serum were collected from 40 patients with CRC, with serum obtained from 23 hospitalized non-cancer controls. Sphingolipids were quantified by UHPLC–MS/MS, while total antioxidant capacity, total oxidant status, and oxidative stress index characterized systemic redox status. Paired lipidomics revealed coordinated remodeling with increased S1P-associated measures, selective ceramide depletion, and elevated S1P-to-ceramide ratios. Multivariate analyses did not identify stable discrete lipidomic subgroups and revealed variation consistent with a continuum-like organization within the measured sphingolipid feature space. In separate principal component analyses, ratio-derived variables showed a more concentrated low-dimensional covariance structure than absolute lipid concentrations. Circulating sphingolipids showed limited correspondence with tumor-local remodeling, whereas oxidative stress markers showed strong apparent discrimination between CRC and hospitalized non-cancer controls, although this finding may be affected by residual confounding. TCGA–GTEx analyses provided complementary pathway-level transcriptomic context, while anatomically resolved analysis of 374 TCGA tumors identified 3376 genes significantly associated with colorectal anatomical position, including six sphingolipid-related genes. Overall, these exploratory findings support a conceptual CRC Metabolic Continuum while requiring validation in larger, independent cohorts.

## 1. Introduction

Colorectal cancer (CRC) is one of the leading causes of cancer-related morbidity and mortality worldwide, accounting for more than 1.9 million new cases and nearly one million deaths annually. Despite substantial advances in screening, surgery, systemic therapy, and molecular classification, CRC remains a biologically heterogeneous disease with marked variability in clinical behavior, treatment response, and patient outcomes. This heterogeneity cannot be explained solely by genetic and epigenetic alterations. It also reflects extensive metabolic reprogramming that enables tumor cells to adapt to changing environmental conditions throughout colorectal carcinogenesis [1,2,3,4].

Metabolic adaptation is now recognized as a fundamental feature of cancer biology. Interconnected metabolic networks coordinate energy production, biosynthesis, inflammatory signaling, cellular differentiation, and interactions with the tumor microenvironment. Their remodeling allows malignant cells to sustain proliferation under nutrient limitation, hypoxia, oxidative stress, and immune pressure, thereby contributing to tumor progression and therapeutic resistance [5,6,7,8].

Redox homeostasis is an integral component of this adaptation. Reactive oxygen species (ROS), once regarded primarily as toxic by-products of aerobic metabolism, are now recognized as signaling mediators influencing proliferation, apoptosis, mitochondrial function, inflammation, and metabolic plasticity. During malignant transformation, persistent oxidative pressure may induce compensatory antioxidant responses and establish a redox state that supports tumor growth while limiting excessive oxidative damage [1,5,6,7,8,9].

This relationship is particularly relevant to lipid metabolism. Lipids function not only as structural components of cellular membranes but also as signaling mediators involved in inflammation, mitochondrial homeostasis, autophagy, and programmed cell death. Among these lipid classes, sphingolipids are especially relevant because their metabolism is closely linked to redox regulation and signaling pathways governing cellular survival and adaptation [10,11,12,13,14,15].

Ceramides, sphingosine, sphingosine-1-phosphate (S1P), and their metabolic intermediates form an interconnected network regulating apoptosis, proliferation, differentiation, autophagy, inflammation, angiogenesis, immune responses, and cellular stress adaptation. Their biological effects depend not only on individual metabolites but also on relationships among interconnected branches of sphingolipid metabolism. This concept is commonly described as the sphingolipid rheostat, in which the balance between ceramide- and S1P-associated signaling contributes to cell-fate regulation [10,11,12,13,14,15,16,17,18].

Redox homeostasis and sphingolipid metabolism are reciprocally interconnected. ROS can modulate enzymes involved in sphingolipid synthesis, remodeling, and degradation, whereas sphingolipid mediators influence mitochondrial function, antioxidant defenses, inflammatory signaling, and redox-sensitive transcriptional pathways. Dysregulation of these interactions may contribute to malignant phenotypes, including sustained proliferation, resistance to apoptosis, immune evasion, and treatment resistance [12,13,14,15,16,17,18,19,20].

Despite growing interest in sphingolipid biology in CRC, its organization in human tumors remains incompletely understood. Most studies have examined individual metabolites, selected metabolic enzymes, or isolated signaling pathways, providing only a partial view of a pathway characterized by coordinated interactions among multiple metabolites and regulatory processes. Moreover, tissue lipidomics and circulating biomarkers represent distinct biological compartments, and their relationship to systemic redox status and broader molecular programs across the colorectum remains insufficiently characterized [16,17,18,19,20,21,22,23].

Advances in quantitative lipidomics, multivariate modeling, and large-scale transcriptomic profiling now permit these questions to be addressed from a systems perspective. Integrating biochemical measurements with transcriptomic information can provide complementary evidence regarding whether tumor-associated lipid remodeling occurs in isolation or is embedded within broader molecular patterns in CRC [21,22,23,24,25,26,27]. Importantly, transcriptomic data provide an independent molecular context rather than direct validation of lipid concentrations or metabolic flux.

Against this background, we hypothesized that sphingolipid remodeling in CRC represents a coordinated biological process rather than a collection of independent metabolic abnormalities. We addressed this hypothesis through a three-layer analytical framework. First, quantitative UHPLC–MS/MS profiling of patient-matched tumor and adjacent non-neoplastic tissues was used to characterize local sphingolipid remodeling while minimizing interindividual biological variability. Second, circulating oxidative stress markers were analyzed alongside serum sphingolipid profiles to characterize a distinct systemic redox and metabolic dimension. Third, these biochemical observations were examined in an independent molecular context using TCGA and GTEx transcriptomic data. Tumor-associated expression of predefined sphingolipid-pathway genes was evaluated against normal colorectal tissue, and a complementary anatomically resolved analysis of TCGA tumors assessed broader transcriptomic variation across the colorectal axis [24,25,26,27,28,29,30,31].

These complementary layers were integrated within a systems biology framework encompassing local tumor metabolism, systemic redox status, and large-scale molecular organization. Rather than seeking only individual lipid biomarkers, we aimed to determine whether CRC-associated sphingolipid remodeling within the measured feature space was better described by discrete metabolic patterns or by a graded, continuum-like organization, and to examine these findings in an independent transcriptomic context. This exploratory framework was intended to characterize metabolic heterogeneity in CRC and generate hypotheses for subsequent validation in larger, independent cohorts.

## 2. Materials and Methods

### 2.1. Patients

The study was conducted in accordance with the Declaration of Helsinki and approved by the Ethics Committee of the Medical University of Bialystok, Poland (APK 002/320/2021). Written informed consent was obtained from all participants.

Forty adult patients with primary colorectal cancer (CRC) scheduled for surgical resection were prospectively enrolled. CRC diagnosis was established by colonoscopy with histopathological confirmation, and preoperative staging was performed using computed tomography. The comparison group consisted of 23 individuals without evidence of colorectal cancer who were hospitalized for non-neoplastic conditions. All participants were of European ancestry.

Peripheral venous blood samples were collected preoperatively in patients with CRC or at the beginning of hospitalization in controls. All samples were obtained before any oncological treatment. In patients with CRC, paired tumor tissue and adjacent macroscopically non-cancerous colorectal mucosa were collected during surgery. Tissue origin was histologically verified. Samples were snap-frozen in liquid nitrogen and stored at −80 °C until analysis. Tumors were classified according to the AJCC 8th edition TNM system.

### 2.2. Sphingolipid Quantification

*Sphingolipid* species were quantified using ultra-high-performance liquid chromatography coupled with tandem mass spectrometry (UHPLC–MS/MS), as previously described by Blachnio-Zabielska et al. [24] and applied in our earlier colorectal cancer study [14,15], with minor matrix-specific adaptations.

For tissue analysis, approximately 20 mg of tissue was homogenized and subjected to organic solvent extraction in the presence of internal standards. The internal standard panel comprised Sph-d7, SPA-d7, S1P-d7, C15:0-d7-Cer, C16:0-d7-Cer, C18:1-d7-Cer, C18:0-d7-Cer, 17C/20:0-Cer, C24:1-d7-Cer, and C24-d7-Cer (Avanti Polar Lipids, Alabaster, AL, USA). For serum analysis, extraction was performed directly from 50 μL aliquots without prior homogenization.

Following extraction, the organic extracts were evaporated, and the residues were reconstituted in the organic mobile phase consisting of methanol containing 2 mm ammonium formate and 0.1% formic acid. Measurements were performed using a TSQ Altis Plus triple quadrupole mass spectrometer coupled to a Vanquish UHPLC system (Thermo Fisher Scientific, Waltham, MA, USA). Chromatographic separation was achieved using a Zorbax SB-C8 column (2.1 × 150 mm, 1.8 μm particle size; Agilent Technologies, Santa Clara, CA, USA).

Analytes were detected using electrospray ionization in selected reaction monitoring (SRM) mode. Sphingosine-1-phosphate (S1P) was analyzed in negative ion mode, whereas the remaining sphingolipids were measured in positive ion mode. Quantification was performed using analyte-specific external calibration curves, with internal standards added to the samples prior to extraction to account for variability during sample preparation and instrumental analysis.

In the original analytical validation of the LC–MS/MS methodology, analyte-specific limits of detection ranged from 2.4 to 5.2 fmol on-column. Extraction recoveries ranged from 85% to 95%, while intra-assay and inter-assay coefficients of variation ranged from 0.1% to 1.1% and from 0.3% to 1.9%, respectively [24]. Analyte-specific limits of quantification (LOQs) applicable to the measurements performed in the present study could not be reliably recovered from the archived analytical documentation and were therefore not retrospectively estimated or reported.

The targeted tissue sphingolipid analysis comprised 21 variables: 12 absolute measures and 9 derived S1P-to-ceramide ratios. The absolute measures included sphingosine (Sph), sphinganine (SPA), sphingosine-1-phosphate (S1P), C14-Cer, C16-Cer, C18:1-Cer, C18-Cer, C20-Cer, C22-Cer, C24:1-Cer, C24-Cer, and total ceramide (Tot-Cer). Tissue sphingolipid concentrations were normalized to tissue mass and expressed as pmol/mg tissue, whereas serum sphingolipid concentrations were expressed as pmol/mL. S1P-to-ceramide ratios were dimensionless.

### 2.3. Oxidative Stress Assessment

Serum oxidative status was evaluated by measuring total antioxidant capacity (TAC), total oxidant status (TOS), and the oxidative stress index (OSI) using validated automated colorimetric methods developed by Erel [25,26]. Total antioxidant capacity was determined according to the ability of serum antioxidants to neutralize the ABTS• + (2,2′-azino-bis(3-ethylbenzothiazoline-6-sulfonate)) radical cation, with results expressed as Trolox equivalents [25]. Total oxidant status was quantified based on the oxidation of ferrous ions (Fe^2+^) to ferric ions (Fe^3+^) by oxidant molecules present in the samples, followed by colorimetric detection using xylenol orange, with results expressed as hydrogen peroxide equivalents [26]. The TOS assay was performed in triplicate for each sample to improve analytical precision.

TAC and TOS values used in the final analyses were expressed in µmol/L. The oxidative stress index (OSI), representing the balance between total oxidant burden and antioxidant defense, was calculated as the ratio of TOS to TAC according to the following equation [26]: OSI = (TOS/TAC) × 100.

TAC, TOS, and OSI were used to characterize systemic redox status as a complementary analytical layer distinct from local tumor sphingolipid remodeling. These circulating measures were interpreted as host-level oxidative-stress indicators and not as direct measures of intratumoral redox status or sphingolipid metabolism [4,9,10,11,12,13].

### 2.4. Statistical and Integrative Analyses

Tumor-associated sphingolipid remodeling was evaluated using paired differences between tumor tissue and matched adjacent non-neoplastic mucosa [Δ(T − N)]. Nonparametric comparisons were performed using the Wilcoxon signed-rank test for paired data and the Mann–Whitney U test for independent groups, as appropriate. For paired tumor–normal comparisons, effect size was quantified using the Wilcoxon rank-biserial correlation coefficient, calculated from the positive and negative signed-rank sums as r_rb_ = (W_+_ − W_−_)/(W_+_ + W_−_). Positive values indicate higher levels in tumor tissue relative to matched non-neoplastic mucosa, whereas negative values indicate lower levels. Multiple comparisons were controlled using the Benjamini–Hochberg false discovery rate (FDR) procedure.

To characterize the organization of tissue sphingolipid remodeling, multivariate analyses were performed on the 21-variable feature set in 40 paired tumor–normal samples. Variables were standardized by z-score transformation before multivariate analysis. Principal component analysis (PCA) was used to identify the dominant axes of variation. PCA was additionally performed separately for absolute lipid measures and S1P-to-ceramide ratio-derived variables to compare the internal covariance structure of these feature spaces. The proportion of variance explained by PC1 in these separate analyses was interpreted as within-block variance concentration rather than as the proportion of a common PC1 independently predicted by either feature block. Because S1P-to-ceramide ratios are mathematically derived from their component metabolites, comparisons between ratio-derived and absolute feature spaces were considered descriptive rather than independent predictive tests.

To evaluate whether the dominant interaction-based structure was preserved after dimensional reduction, a predefined reduced three-ratio panel was compared with the full nine-ratio feature set. PCA was performed separately for the full and reduced ratio panels, and the proportion of within-panel variance captured by PC1 was recorded for each feature set. Concordance of the reduced representation with the dominant axis derived from the full ratio panel was quantified using the coefficient of determination (R^2^), and its internal stability was assessed using leave-one-out cross-validation (LOOCV). In addition, the first principal components obtained independently from the reduced and full panels were compared directly to assess preservation of the dominant covariance structure.

Redundancy among ratio-derived variables was assessed using pairwise correlations and multicollinearity diagnostics. For each ratio variable, the proportion of variance explained by regression on all remaining ratio variables was quantified as R^2^, and the corresponding variance inflation factor (VIF) was calculated as VIF = 1/(1 − R^2^). Maximum and mean absolute pairwise correlations were also calculated to characterize local and overall redundancy within the ratio feature block. For selected log-transformed S1P-to-ceramide ratios, total variance was additionally decomposed algebraically into numerator, denominator, and covariance contributions according to Var(A − B) = Var(A) + Var(B) − 2Cov(A,B). These analyses were used descriptively to characterize overlapping information arising from the shared mathematical structure of the ratio variables.

Nonlinear dimensionality reduction was performed for exploratory visualization using uniform manifold approximation and projection (UMAP) and t-distributed stochastic neighbor embedding (t-SNE). UMAP was performed with n_neighbors = 12, min_dist = 0.15, Euclidean distance, and random seed 0. t-SNE was performed with perplexity 10, PCA initialization, and random seed 0. These embeddings were used for visualization and were not interpreted as independent evidence of either discrete subgroup structure or continuous biological organization.

Potential discrete lipidomic subgroup structure was evaluated using complementary clustering diagnostics. Silhouette analysis was assessed for k = 2–6 clusters, and the gap statistic was evaluated for k = 1–6 using 20 uniformly generated reference datasets. Gaussian mixture modeling and Hopkins statistics were used as additional assessments of cluster structure and clustering tendency. Extended robustness analyses included repeated Hopkins testing and resampling-based assessments of clustering stability. Given the modest cohort size (*n* = 40), failure to identify stable clusters was interpreted as absence of detectable cluster structure within the measured sphingolipid feature space rather than as evidence excluding biologically meaningful metabolic subgroups.

The stability of the principal component structure was further examined using bootstrap resampling with 1000 iterations and leave-one-out analyses to assess sensitivity to sampling variability and individual observations.

Cross-compartment associations between paired tissue-remodeling indices and matched serum biomarkers were assessed using Spearman rank correlations, with Benjamini–Hochberg FDR correction across the complete tissue–serum correlation matrix.

Cross-compartment multivariate correspondence was additionally explored using partial least squares (PLS) analysis applied to the paired tissue-remodeling and serum feature matrices. Latent scores derived from the two compartments were compared to assess the extent of shared patient-level multivariate structure. Because PLS was used as an exploratory integrative analysis in a modest-sized cohort, the resulting latent-variable correspondence was interpreted descriptively and not as independently validated evidence of biological coupling.

Serum analyses compared 40 patients with CRC with 23 hospitalized non-cancer controls. Associations and discriminatory performance of circulating sphingolipid and oxidative-stress variables were evaluated using correlation analyses, receiver operating characteristic (ROC) analysis, and logistic regression. Multivariable logistic models were evaluated using stratified five-fold cross-validation, and bootstrap procedures were used to estimate uncertainty in ROC performance. ROC analysis was supplemented by Youden-index-based cutoff estimation, graphical calibration assessment, and decision-curve analysis (DCA). Calibration was evaluated by comparing model-predicted probabilities with observed CRC status. DCA was used to estimate net benefit across threshold probabilities ranging from 0.10 to 0.50 for individual serum biomarkers and for models based on lipid variables, oxidative-stress markers, and their combination. Mean and peak net benefit within this threshold range were additionally used for descriptive within-cohort ranking of individual biomarkers. Because the serum comparison group was not prospectively matched to the CRC cohort for relevant demographic and clinical covariates, these analyses were considered exploratory. Accordingly, AUC, calibration, and decision-curve results were interpreted as measures of apparent within-cohort performance rather than as evidence of CRC-specific diagnostic performance or externally validated clinical utility.

Statistical analyses were performed using TIBCO Statistica 14.1 (TIBCO Software Inc., Santa Clara, CA, USA) and Python 3.11 (Python Software Foundation, USA). The reproducibility environment deposited with the analysis code used NumPy 1.26.4, pandas 2.2.2, SciPy 1.13.1, scikit-learn 1.5.1, umap-learn 0.5.6, and statsmodels 0.14.2. All tests were two-sided, and *p* < 0.05 was considered statistically significant unless otherwise specified. Multivariable and machine-learning analyses were considered exploratory and hypothesis-generating.

### 2.5. Independent Transcriptomic Support and Anatomically Resolved Colorectal Axis Analysis

To provide an independent transcriptomic context for sphingolipid-pathway remodeling and to examine whether gene-expression patterns vary across colorectal anatomy, publicly available RNA-sequencing data from The Cancer Genome Atlas (TCGA) and the Genotype-Tissue Expression (GTEx) project were analyzed. Uniformly processed gene-expression data were obtained from the UCSC Xena Toil RNA-seq Recompute Compendium, thereby reducing technical variability associated with differences in alignment and expression-quantification pipelines. The dataset comprised 308 non-neoplastic colon samples from GTEx (141 sigmoid and 167 transverse colon), 288 primary colon adenocarcinomas from TCGA-COAD, and 92 primary rectal adenocarcinomas from TCGA-READ. Gene-expression values were analyzed as log_2_(TPM + 1).

Gene identifiers were harmonized using the accompanying annotation resources, with Ensembl identifiers mapped to official HGNC gene symbols. Targeted analyses focused on evaluable genes representing major components of sphingolipid metabolism, including de novo sphingolipid synthesis, ceramide generation and remodeling, sphingosine-1-phosphate metabolism, and S1P receptor signaling. Expression patterns were compared between GTEx normal colorectal tissue, TCGA-COAD, TCGA-READ, and pooled CRC samples. CRC-associated transcriptional differences were summarized at both individual-gene and pathway levels. Because transcript abundance does not directly measure enzyme activity, metabolic flux, or lipid concentrations, these analyses were interpreted as an independent transcriptomic context rather than direct validation of the lipidomic findings.

To quantify coordinated transcriptional remodeling of the sphingolipid rheostat, a sample-level sphingolipid rheostat remodeling score (SRRS) was calculated from the integrated expression profile of rheostat-associated genes. Expression of each included gene was standardized as a z-score across analyzed samples, and each gene was assigned a directional weight according to the sign of its CRC-versus-normal dysregulation. The SRRS for each sample was calculated as the mean of the products of standardized gene expression and the corresponding directional weights. SRRS distributions were compared between normal colorectal tissue, TCGA-COAD, and TCGA-READ using Mann–Whitney U tests.

A complementary anatomically resolved analysis was performed using RNA-sequencing profiles from 374 primary TCGA colorectal tumors with detailed anatomical annotation. Tumors were ordered along a predefined cecum-to-rectum anatomical axis comprising cecum, ascending colon, hepatic flexure, transverse colon, splenic flexure, descending colon, sigmoid colon, and rectum. The single rectosigmoid junction tumor was assigned to the sigmoid–rectal transition within this anatomical ordering.

Genome-wide transcriptomic organization was explored using principal component analysis applied to 19,885 genes. Associations between principal components and anatomical position were assessed using Spearman correlation. Gene-wise regression models were subsequently used to identify gene-expression patterns associated with position along the colorectal axis, with Benjamini–Hochberg false discovery rate correction for multiple testing. Particular emphasis in the integrative interpretation was placed on sphingolipid-related genes, whereas the broader genome-wide analysis of position-associated expression was considered complementary and is reported in greater detail in the Supplementary Materials. Because individual anatomical subsites were unevenly represented, these analyses were interpreted as population-level associations between gene expression and colorectal position rather than precise estimates of expression states at each anatomical subsite.

Although Toil processing provides a uniform computational framework for TCGA and GTEx RNA-sequencing data, residual cohort- and source-related effects cannot be completely excluded. Accordingly, TCGA–GTEx comparisons were interpreted as supportive transcriptomic evidence rather than direct biochemical validation.

## 3. Results

### 3.1. Patient Cohort and Paired Sphingolipid Remodeling

The study included 40 patients with colorectal cancer (CRC) who underwent paired analysis of tumor tissue and adjacent non-neoplastic mucosa (22 men and 18 women; mean age, 71.8 ± 10.0 years). Most tumors were moderately differentiated (G2, *n* = 36). TNM stages comprised stage I (*n* = 2), stage II (*n* = 16), stage III (*n* = 17), and stage IV (*n* = 5). Detailed clinicopathological characteristics are provided in Supplementary Table S1.

**Figure 1:**
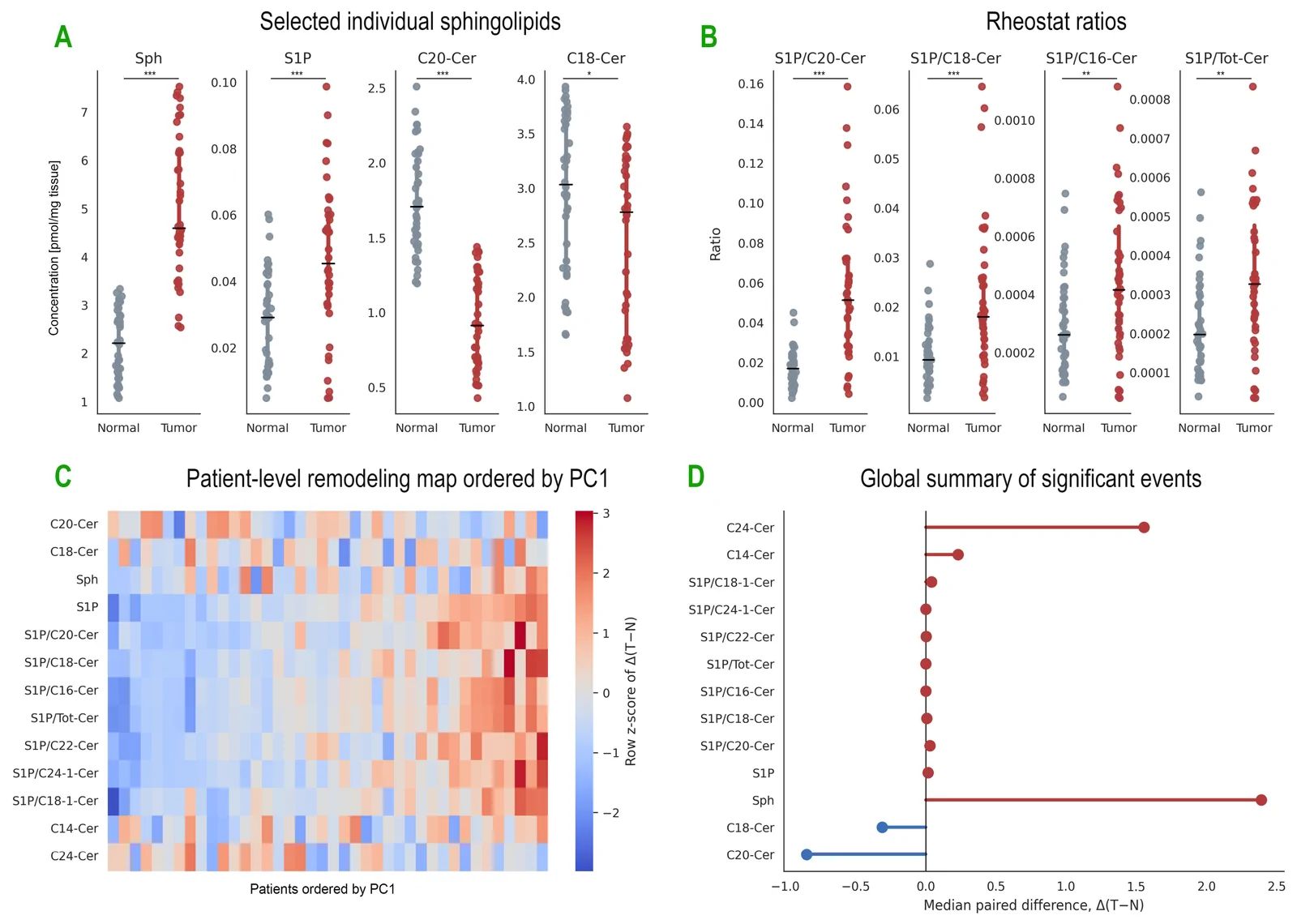
Coordinated sphingolipid remodeling in colorectal cancer tumors. (**A**) Paired comparison of selected sphingolipid species between tumor tissue and adjacent normal mucosa. Tissue sphingolipid concentrations were normalized to tissue mass and expressed as pmol/mg of tissue. Each point represents one patient (*n* = 40). Horizontal bars indicate median values and interquartile ranges. Tumors show increased levels of sphingosine (Sph) and sphingosine-1-phosphate (S1P), together with reduced levels of several ceramide species, most prominently C20-ceramide. Statistical significance was assessed using the paired Wilcoxon signed-rank test with Benjamini–Hochberg FDR correction: * = FDR < 0.05; ** = FDR < 0.01; *** = FDR < 0.001. (**B**) Paired comparison of key sphingolipid rheostat ratios. Multiple S1P-to-ceramide ratios are increased in tumor tissue, indicating a systematic shift in the relative balance between S1P and ceramide species. (**C**) Heatmap of patient-level tumor–normal differences (ΔT − N) across significant sphingolipid variables. Values are row-standardized (z-scores) to highlight relative remodeling patterns. Patients are ordered along the first principal component (PC1) for visualization, showing a graded cross-sectional distribution of metabolic states within the measured sphingolipid feature space. PC1-based ordering does not imply temporal progression. (**D**) Summary of median paired differences for significant sphingolipid variables arranged in mechanistic order. Negative values indicate reduced levels in tumors (blue), whereas positive values indicate increased levels (red). The combined pattern reflects coordinated remodeling of the sphingolipid network characterized by selective reductions in ceramide species and increased S1P-associated rheostat measures.

Paired tumor–normal comparisons revealed extensive remodeling of the sphingolipid profile (Figure 1A–D). Following false discovery rate (FDR) correction, 13 of 21 analyzed sphingolipid-related variables remained significantly altered. Tumor tissue showed significantly higher levels of sphingosine (Sph) and sphingosine-1-phosphate (S1P). Sph showed one of the strongest paired effects, with higher tumor levels observed in 39 of 40 paired samples (97.5%; FDR < 0.0001; Wilcoxon rank-biserial r_rb_ = 0.995), while S1P was also significantly increased (FDR = 0.0001; r_rb_ = 0.710). Multiple S1P-to-ceramide ratios were increased, including S1P/C20-Cer (FDR < 0.0001; r_rb_ = 0.937) and S1P/C18-Cer (FDR < 0.0001; r_rb_ = 0.776).

In contrast, C20-Cer showed the strongest tumor-associated decrease, with lower tumor levels observed in all 40 paired samples (40/40, 100%; FDR < 0.0001; Wilcoxon rank-biserial r_rb_ = −1.000), while C18-Cer was also significantly reduced (FDR = 0.0335; r_rb_ = −0.417). Complete paired statistics, FDR-adjusted *p*-values, Wilcoxon rank-biserial effect sizes, and confidence intervals are provided in Supplementary Tables S2 and S3.

### 3.2. Continuum-like Organization of Tumor Sphingolipid Remodeling

**Figure 2:**
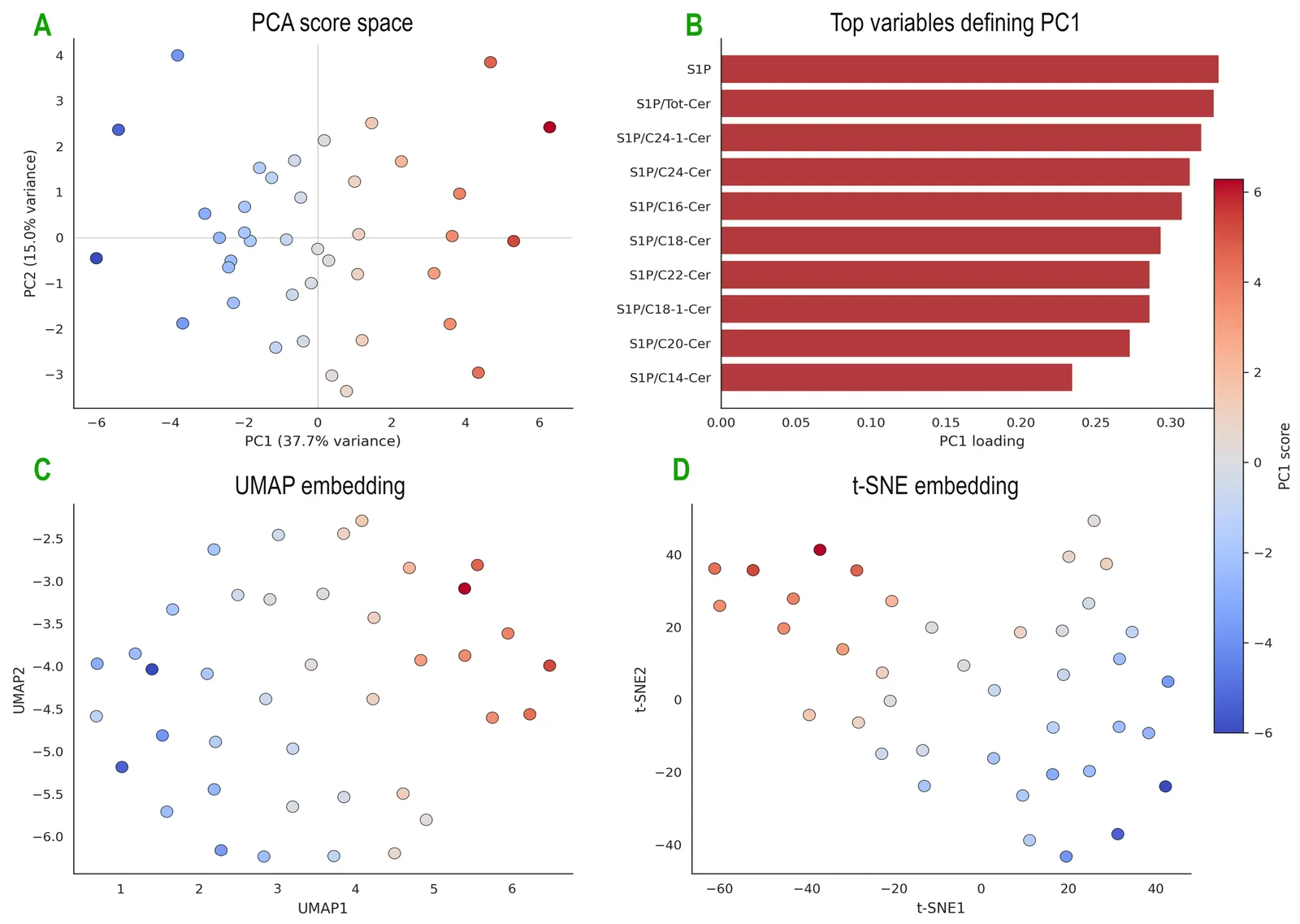
Sphingolipid remodeling in colorectal cancer is compatible with a continuum-like metabolic landscape. (**A**) Principal component analysis (PCA) of paired tumor–normal sphingolipid differences [Δ(T − N)] across colorectal cancer samples. Each point represents one tumor, colored according to its position along the dominant metabolic axis (PC1). Samples show a graded distribution along the dominant axis without clearly separated clusters. (**B**) Variables with the strongest contributions to PC1. Interaction-based S1P-to-ceramide ratios contribute prominently to the principal metabolic axis, consistent with a strong contribution of rheostat-related covariance to the dominant pattern of tumor sphingolipid remodeling. (**C**) UMAP and (**D**) t-SNE embeddings of the same dataset recover a similar sample geometry, compatible with a continuum-like organization within the measured sphingolipid feature space.

Principal component analysis identified a dominant axis of variation across the 21-variable tissue sphingolipid feature space. UMAP and t-SNE were used as complementary exploratory visualizations of the same dataset and showed broadly similar sample geometry without clearly separated groups (Figure 2A–D).

The observed sample distribution was therefore consistent with a continuum-like organization along the dominant axes of sphingolipid variation. However, given the modest cohort size (*n* = 40), these analyses cannot distinguish a genuine biological continuum from limited statistical power to resolve smaller, overlapping, or weakly separated metabolic subgroups. Accordingly, the findings should not be interpreted as evidence that biologically meaningful sphingolipid subtypes do not exist in CRC.

To assess whether this organization reflected discrete metabolic subgroups or continuous variation, complementary clustering diagnostics were applied. Silhouette analysis, gap statistics, Gaussian mixture modeling, and Hopkins testing provided no evidence of stable lipidomic subclasses (Supplementary Table S6). Consistent sample organization was observed across PCA, UMAP, and t-SNE representations (Figure 2A–D). Together, these analyses showed a graded distribution of metabolic profiles along the dominant axes of variation without detectable separation into stable lipidomic clusters, a pattern compatible with a continuum-like organization within the measured feature space.

### 3.3. Ratio-Derived Metrics Show a More Concentrated Dominant Axis of Variation

To examine how absolute sphingolipid concentrations and interaction-based rheostat metrics relate to the dominant metabolic axis, complementary PCA and regression-based analyses were performed. In separate PCA models, PC1 accounted for 22.6% of the total variance in the absolute-lipid feature space and 69.5% in the S1P-to-ceramide ratio feature space. These percentages represent the concentration of variance within two separately analyzed feature spaces and should not be interpreted as the proportion of a common PC1 independently explained by either feature class.

**Figure 3:**
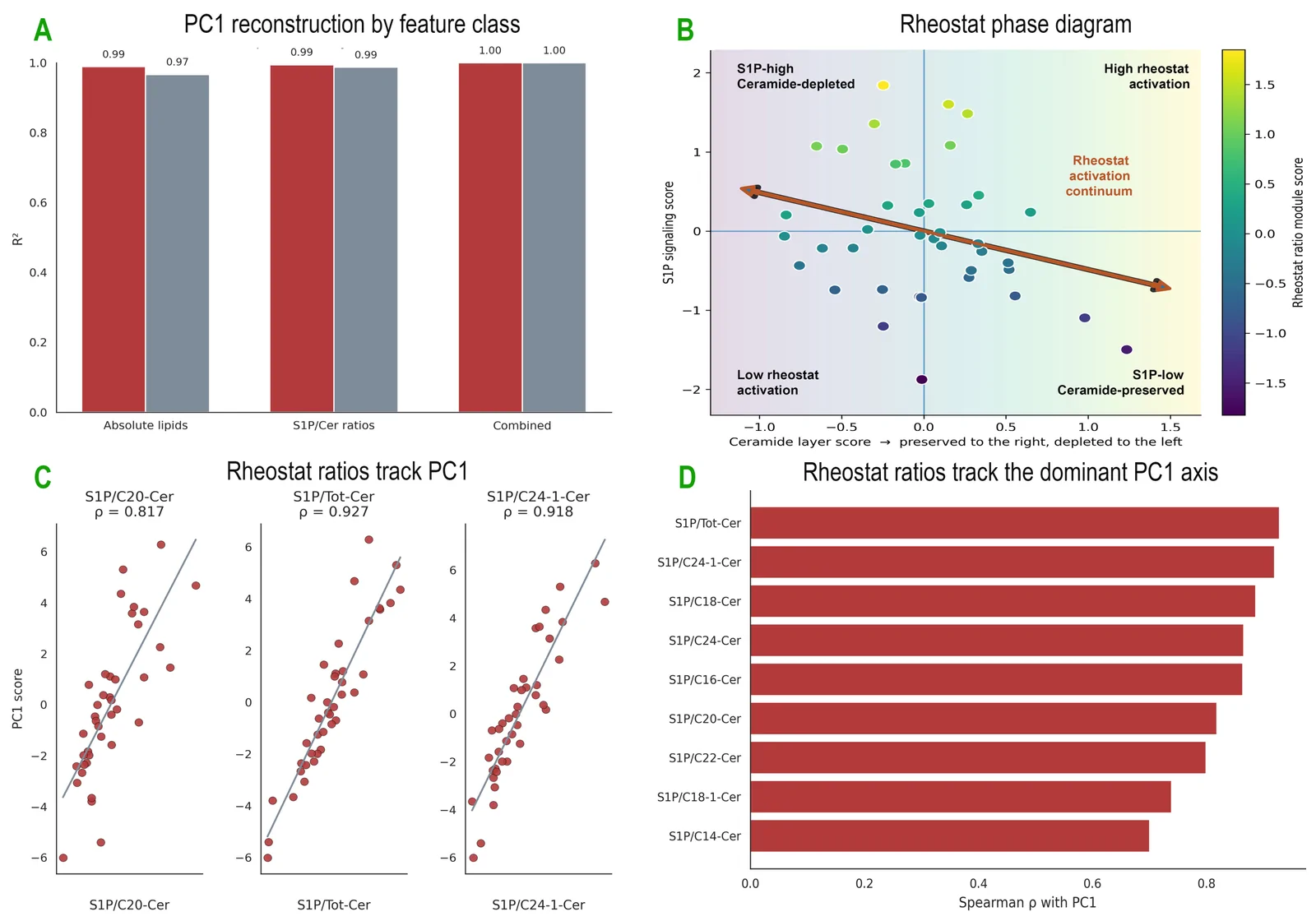
Interaction-based rheostat metrics define the dominant metabolic axis. (**A**) Comparison of regression models reconstructing the dominant tissue metabolic axis (PC1), derived from the complete standardized sphingolipid feature matrix, using absolute lipid concentrations, S1P-to-ceramide ratios, or both feature classes combined. Because these feature classes contributed to the PCA defining PC1, the near-saturated R^2^ values (0.97–1.00) reflect internal reconstruction of the same multivariate structure and should not be interpreted as independent predictive performance. (**B**) Rheostat phase diagram of tumor sphingolipid remodeling; each point represents an individual tumor sample positioned according to composite scores reflecting the ceramide layer (*x*-axis) and S1P signaling module (*y*-axis). The diagram illustrates the cross-sectional distribution of sphingolipid rheostat states across colorectal tumors, ranging from ceramide-preserved, lower-S1P states to S1P-dominant, ceramide-depleted states. The double-headed arrow indicates the empirical direction of variation estimated from the regression line across tumors and does not imply temporal progression. Color intensity reflects the composite S1P-to-ceramide ratio measure. (**C**) Descriptive relationships between PC1 and representative rheostat variables, including S1P/C20-Cer, S1P/Total-Cer, and S1P/C24:1-Cer. (**D**) Spearman correlations between PC1 and S1P-to-ceramide ratios across the denominator panel. Because ratio-derived variables contributed to the feature matrix defining PC1, the associations in panels (**C**,**D**) describe the covariance structure of the dataset and should not be interpreted as independent predictive evidence.

Complementary regression models assessed how effectively absolute lipid concentrations, S1P-to-ceramide ratios, or their combination could reconstruct PC1 derived from the complete standardized sphingolipid feature matrix (Figure 3A; Supplementary Table S7). Both feature classes showed strong correspondence with this dominant axis, with near-saturated R^2^ values across all feature blocks (0.97–1.00). These high values further illustrate the internal and partly circular nature of the reconstruction, because the analyzed feature classes were themselves included in the PCA used to define PC1, and should therefore not be interpreted as independent predictive performance. Moreover, the shared S1P numerator and mathematical coupling between ratios and their component metabolites may contribute to their strong covariance structure.

Consistent with this interpretation, PC1 was strongly associated with ΔS1P and several S1P-to-ceramide ratios, including S1P/Total-Cer, S1P/C24:1-Cer, S1P/C24-Cer, S1P/C16-Cer, and S1P/C18-Cer (Figure 3C,D). These associations are therefore interpreted descriptively as features of the covariance structure underlying the dominant metabolic axis rather than as independent evidence that ratio-derived metrics outperform absolute lipid concentrations.

Variance decomposition of representative S1P-to-ceramide ratios, including the relative contributions of S1P, ceramide, and covariance components, is provided in Supplementary Table S8. Analysis of a reduced three-ratio rheostat panel showed that the principal interaction-based structure was retained after dimensional reduction (Supplementary Table S9). Multicollinearity analysis further identified substantial redundancy among several S1P-to-ceramide ratios (Supplementary Table S14), consistent with their shared mathematical structure.

### 3.4. C20-Ceramide Shows Consistent Tumor-Associated Remodeling

C20-Cer showed a consistent tumor-associated reduction (median Δ = −0.85) with relatively low interindividual variability. Its contribution to PC1 was modest, whereas the corresponding S1P/C20-Cer ratio showed a strong descriptive association with the dominant metabolic axis (Spearman *ρ* = 0.817) (Figure 3C).

Thus, C20-Cer showed a consistent pattern of tumor-associated reduction across paired samples. The strong association between S1P/C20-Cer and PC1 further reflects the coordinated covariance structure of S1P and ceramide-related variables within the multivariate dataset; because ratio-derived variables contributed to the PCA defining PC1, this association should not be interpreted as independent evidence of superior predictive performance.

### 3.5. Limited Correspondence Between Tissue and Circulating Metabolic Profiles

**Figure 4:**
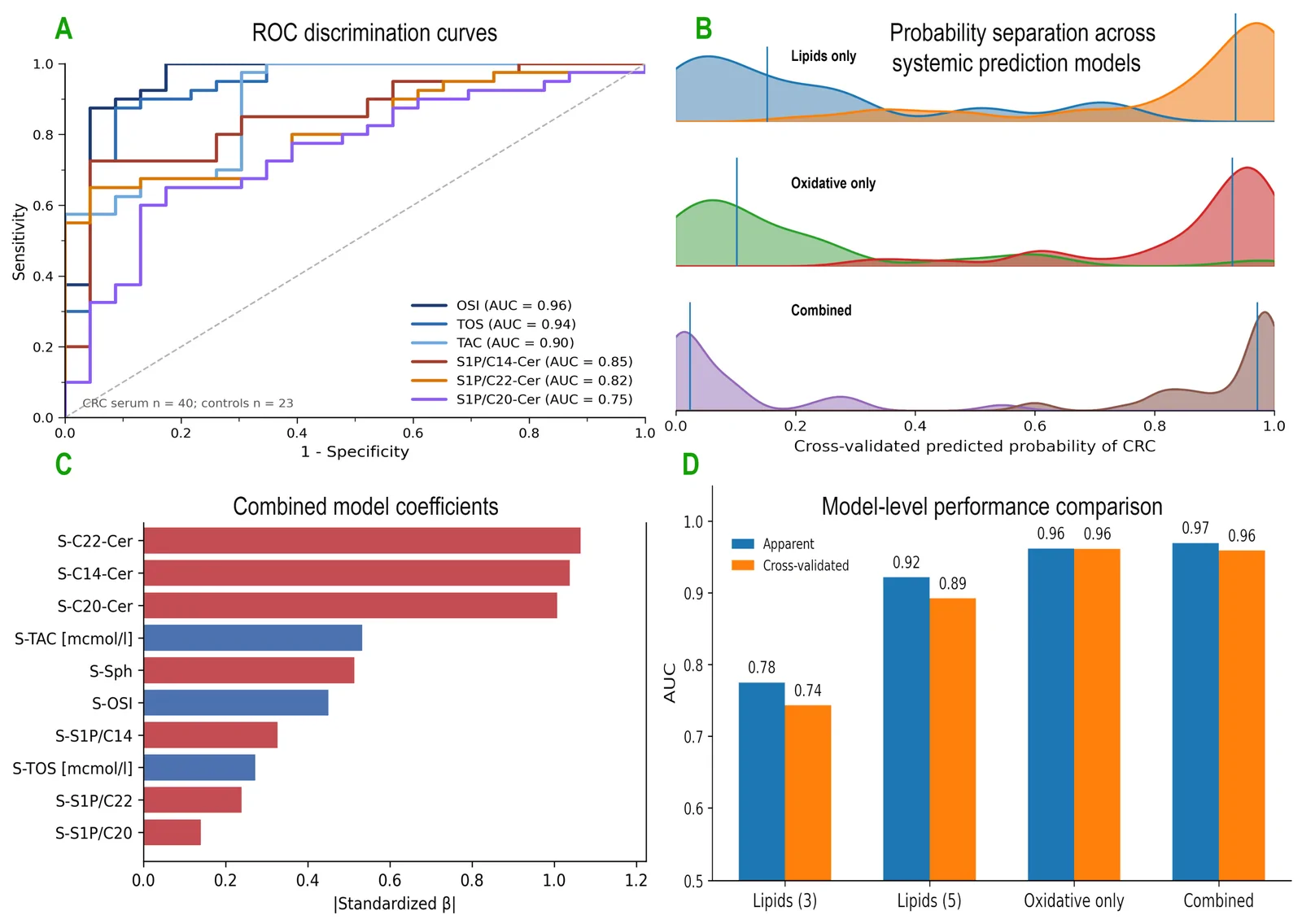
Circulating sphingolipid and oxidative signals distinguish patients with CRC from hospitalized non-cancer controls across complementary modeling frameworks. (**A**) Receiver operating characteristic (ROC) curves for the strongest within-cohort serum discriminators of colorectal cancer, including oxidative stress markers (OSI, TOS, TAC) and selected sphingolipid ratios (S1P/C14-Cer, S1P/C22-Cer, S1P/C20-Cer). (**B**) Distribution of cross-validated predicted probabilities of colorectal cancer generated by lipid-based, oxidative, and combined systemic models. Density plots illustrate the separation between CRC patients and controls, with vertical ticks indicating group medians. (**C**) Absolute standardized coefficients from the combined logistic model, showing contributions from both oxidative markers and sphingolipid variables, with no single feature class exclusively determining model structure. (**D**) Model-level performance comparison across minimal lipid (3-variable), expanded lipid (5-variable), oxidative-only, and combined predictor sets. Lipid model performance improves with feature expansion, while oxidative markers provide consistently strong discrimination. The combined model showed the highest apparent performance, consistent with complementary information from sphingolipid and oxidative variables within the present cohort. Blue bars indicate apparent AUC values, and orange bars indicate cross-validated AUC values.

**Figure 5:**
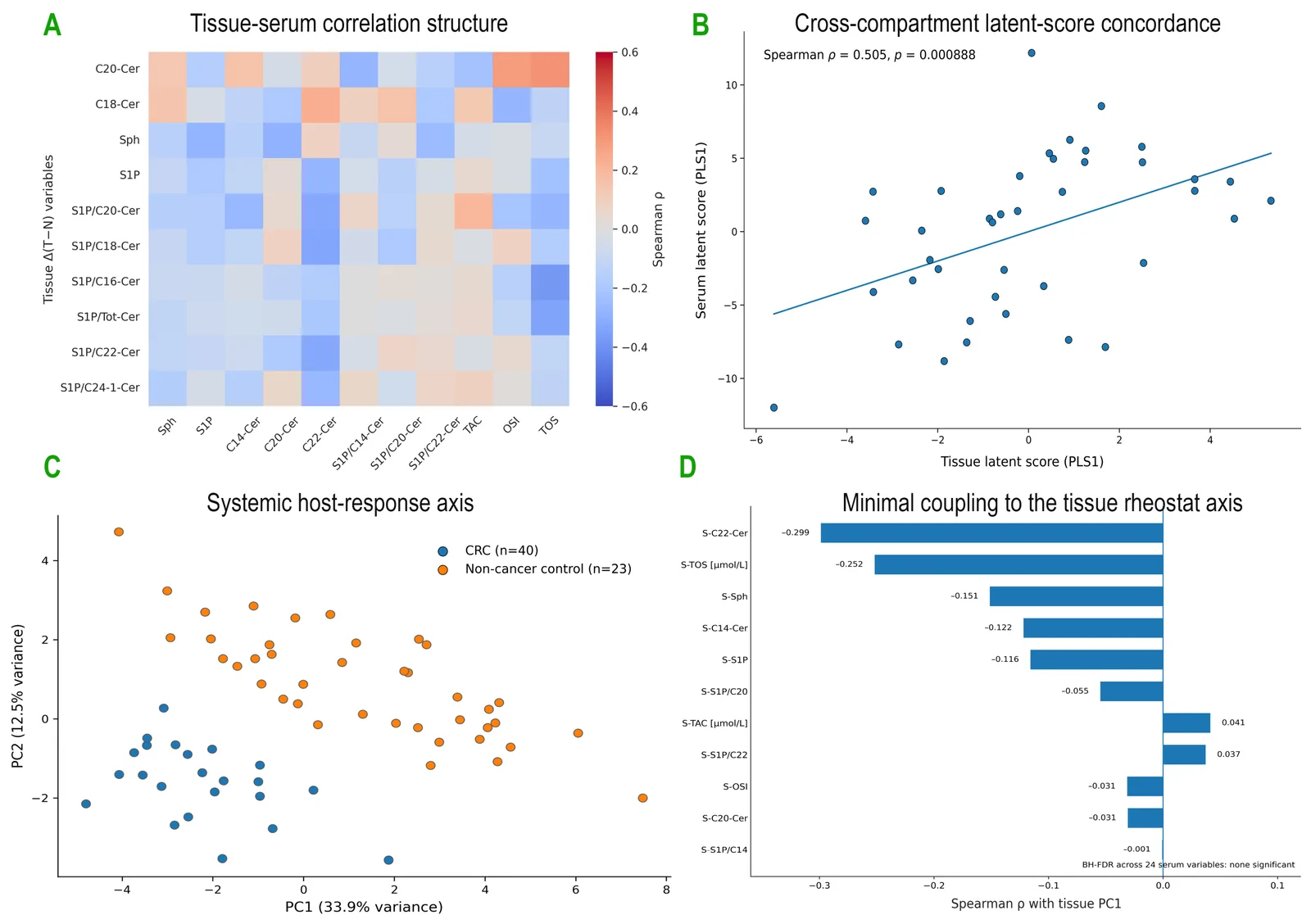
Limited coupling between tissue sphingolipid remodeling and systemic host-response signals. (**A**) Spearman correlation heatmap showing associations between paired tumor–normal differences in tissue sphingolipids (ΔT − N) and circulating serum variables, including sphingolipids and oxidative stress markers (TAC and TOS). Correlations were generally low to moderate, indicating limited correspondence between local tumor-associated remodeling and circulating metabolic signals. (**B**) Partial least squares (PLS) analysis of paired tissue and serum sphingolipid profiles from 40 patients with colorectal cancer. Tissue and serum PLS1 scores showed moderate cross-compartment concordance (Spearman *ρ* = 0.505, *p* = 0.000888), indicating a detectable but incomplete shared patient-level structure. (**C**) Principal component analysis (PCA) of serum variables in patients with colorectal cancer (CRC, *n* = 40) and non-cancer controls (*n* = 23). PC1 and PC2 accounted for 33.9% and 12.5% of the total variance, respectively, revealing a dominant systemic axis integrating circulating sphingolipid and oxidative-stress signals and differentiating the overall serum profiles of CRC patients and controls. (**D**) Spearman correlations between selected serum variables and the primary tissue-derived sphingolipid axis (tissue PC1). Associations were weak (|*ρ*| ≤ 0.299), and none remained significant after Benjamini–Hochberg correction across all 24 tested serum variables, supporting limited coupling between individual circulating markers and the tissue sphingolipid rheostat.

The circulating biomarker analyses, including oxidative stress markers, sphingolipid variables, and their combined models, are summarized in Figure 4. Cross-compartment analysis revealed limited correspondence between tumor-associated tissue remodeling and circulating sphingolipid profiles. Although several nominal associations were observed between paired tissue Δ(T − N) values and serum variables, none remained significant after FDR correction (Figure 5A; Supplementary Table S13). Circulating sphingolipid variables also showed minimal association with the dominant tumor metabolic axis.

Multiblock integration further demonstrated limited shared structure between tissue and serum datasets. Partial least squares analysis showed moderate but incomplete cross-compartment correspondence, whereas individual tissue–serum associations remained weak and did not survive multiple-testing correction (Figure 5B,D).

Despite this limited coupling, combined circulating lipid and oxidative-stress variables formed a dominant systemic axis (PC1) explaining 33.9% of serum variance. This axis showed apparent within-cohort discrimination between patients with CRC and hospitalized non-cancer controls (AUC = 0.871). The corresponding separation of serum profiles along the principal component space is illustrated in Figure 5C. Given the unmatched nature and clinical heterogeneity of the control group, this discrimination may reflect both CRC-associated and nonspecific host-related differences and should not be interpreted as CRC-specific diagnostic or screening performance. These findings therefore indicate that the circulating metabolic profile captures systemic variation that is largely distinct from tumor-local sphingolipid remodeling.

### 3.6. Oxidative Stress Markers Show the Strongest Apparent Within-Cohort Discrimination

**Figure 6:**
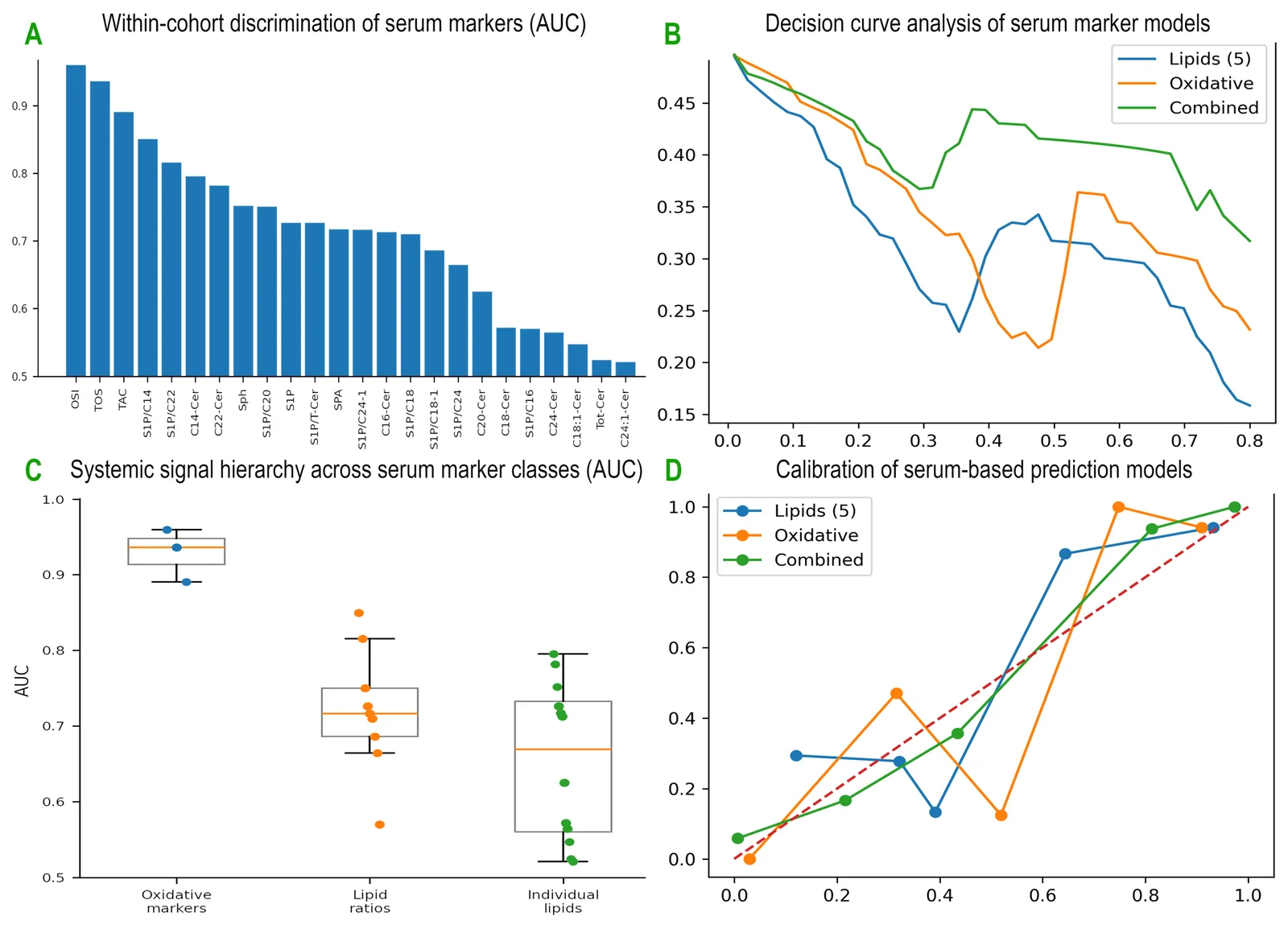
Integrated evaluation of apparent within-cohort discrimination and model behavior across oxidative and sphingolipid biomarkers in colorectal cancer. (**A**) Ranking of serum markers based on receiver operating characteristic (ROC) analysis. Individual circulating markers were ranked according to their area under the receiver operating characteristic curve (AUC) for discrimination between patients with colorectal cancer and hospitalized non-cancer controls. Oxidative stress markers (OSI, TOS, TAC) showed the highest apparent within-cohort discrimination, followed by sphingolipid ratios (S1P/Cer), whereas individual lipid species generally showed lower discriminatory ability. (**B**) Decision curve analysis (DCA) of serum-based models. Net benefit across threshold probabilities is shown for models based on sphingolipids (Lipids), oxidative stress markers (Oxidative), and their combination (Combined). The combined model showed the highest apparent net benefit across a broad range of thresholds within the analyzed cohort, whereas oxidative markers generally showed greater net benefit than lipid-based models alone. (**C**) Distribution of apparent discriminatory performance across serum marker classes. Boxplots show the distribution of AUC values for oxidative stress markers, sphingolipid ratios (S1P/Cer), and individual lipid species. Oxidative markers showed the highest and most consistent apparent discrimination, sphingolipid ratios showed intermediate performance, and individual lipid species showed lower and more variable discrimination. (**D**) Calibration of representative serum-based models. Calibration plots compare predicted and observed event probabilities for models based on sphingolipids (Lipids), oxidative stress markers (Oxidative), and their combination (Combined). The dashed diagonal line indicates perfect calibration. The combined model showed the closest agreement with the ideal line within the analyzed cohort, whereas lipid- and oxidative-only models showed greater deviation across probability ranges. All discrimination, decision-curve, and calibration results are exploratory and reflect performance within the present study cohort. Because the hospitalized non-cancer control group was not prospectively matched to the CRC cohort for demographic and clinical characteristics, these findings should not be interpreted as externally validated CRC-specific diagnostic or screening performance.

Among circulating biomarkers, oxidative stress variables showed the strongest apparent within-cohort discrimination between patients with CRC and hospitalized non-cancer controls. OSI achieved an apparent AUC of 0.96, followed by TOS (AUC = 0.94) and TAC (AUC = 0.90). TAC was significantly reduced in patients with CRC compared with non-cancer controls (Figure 4A and Figure 6A; Supplementary Tables S10 and S11).

These estimates represent apparent discriminatory performance within the present cohort and should not be interpreted as CRC-specific diagnostic or screening performance. Because the control group was not prospectively matched to the CRC group for demographic and clinical characteristics, differences in systemic oxidative-stress markers may be influenced by age, sex, comorbidities, medication use, nutritional status, inflammatory burden, and other host-related factors. Accordingly, the observed differences in TAC, TOS, and OSI are interpreted as systemic redox differences between the study groups rather than as CRC-specific oxidative-stress signatures.

### 3.7. Relationship Between Metabolic Remodeling and Clinicopathological Characteristics

Exploratory analyses were performed to determine whether the principal tissue and circulating metabolic axes were associated with clinicopathological characteristics. No statistically significant associations were observed between the dominant tissue metabolic axis and major clinical variables after correction for multiple testing. Similarly, circulating metabolic axes showed no robust associations with TNM stage or other clinicopathological characteristics.

These findings indicate that no clear relationships between the principal metabolic patterns and conventional clinicopathological characteristics were detectable within the present cohort. However, given the modest sample size and limited representation of individual clinical subgroups, these negative findings should not be interpreted as evidence that such associations are absent. The clinicopathological analyses should therefore be regarded as exploratory.

### 3.8. Sensitivity and Robustness Analyses

Sensitivity analyses were performed to evaluate the stability of the principal findings across alternative analytical specifications. The dominant multivariate structure was stable across complementary dimensionality-reduction approaches, alternative feature sets, and reduced rheostat models. PCA stability was further assessed using 1000 bootstrap iterations and leave-one-out analyses, which supported the robustness of the dominant component structure to sampling variability and individual observations.

Clustering tendency was evaluated using complementary approaches, including silhouette analysis, gap statistics, Gaussian mixture modeling, and repeated Hopkins testing. Additional resampling-based analyses and alternative UMAP specifications did not reveal a reproducible discrete subgroup structure. These findings support the robustness of the observed absence of detectable stable clusters within the analyzed dataset, while not excluding the possibility of biologically relevant subgroups that could not be resolved in this cohort.

Removal of the single detected outlier had virtually no effect on PC1 organization (Spearman *ρ* = 0.999), and a similar overall metabolic structure was observed across metastatic and non-metastatic subgroups.

Because only five rectal cancers were included in the cohort, a separate colon-versus-rectum subgroup analysis was considered statistically underpowered and was therefore not performed. To assess whether the identified sphingolipid remodeling pattern was influenced by broader anatomical tumor location, an additional sensitivity analysis comparing right-sided and left-sided CRC was performed. No statistically significant differences were observed in major tissue or circulating sphingolipid variables, including S1P, C20-Cer, S1P-to-ceramide ratios, or systemic oxidative stress markers. A non-significant trend toward higher tissue S1P levels was observed in right-sided tumors (*p* = 0.089).

### 3.9. TCGA–GTEx Transcriptomic Context for Sphingolipid-Pathway Remodeling

To examine whether CRC-associated transcriptional alterations involved components of sphingolipid metabolism, expression of the predefined sphingolipid-related gene panel was compared between normal colorectal tissue from GTEx and colorectal cancer samples from TCGA, including colon adenocarcinoma (COAD), rectal adenocarcinoma (READ), and the pooled CRC cohort.

**Figure 7:**
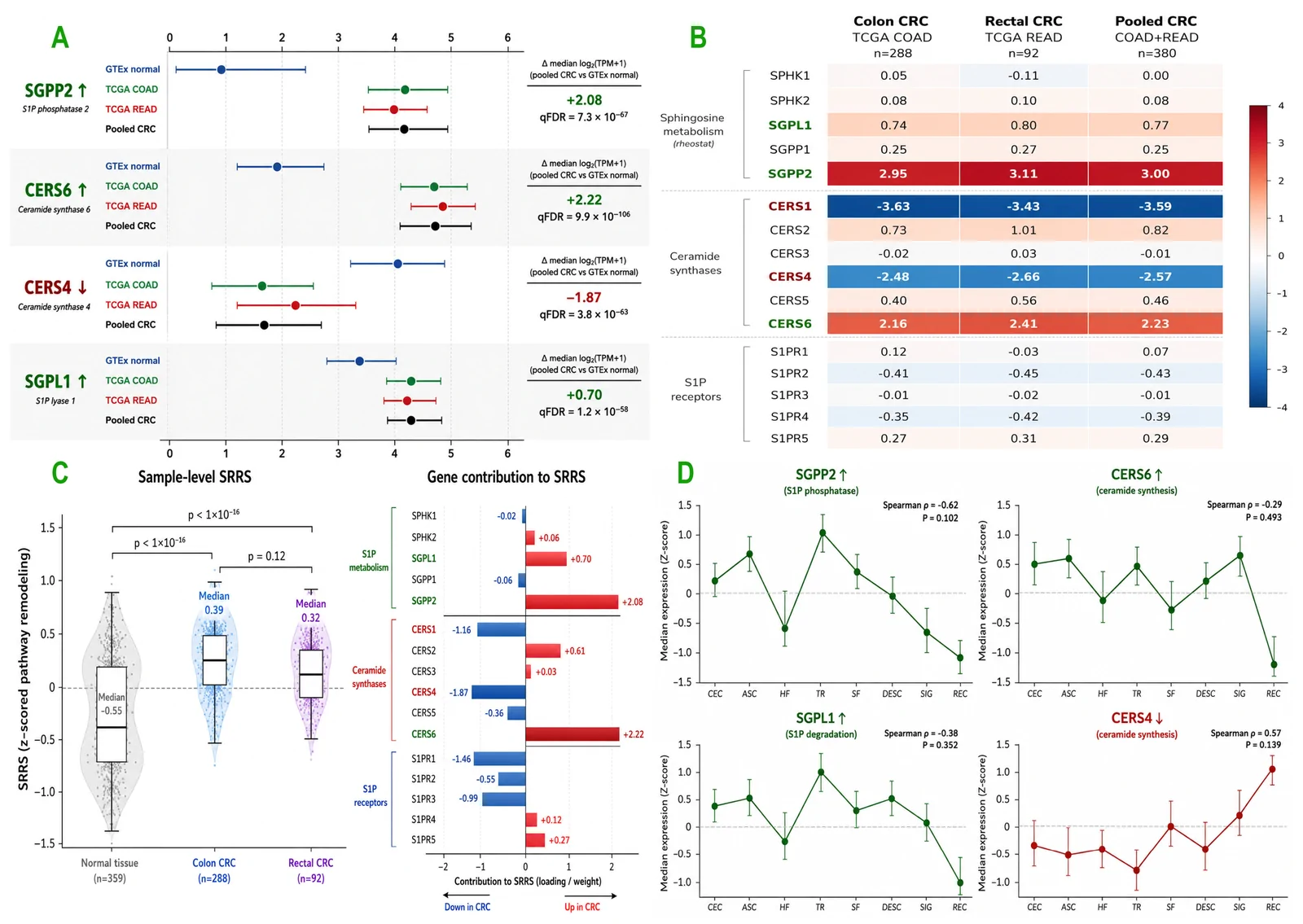
Multi-level characterization of sphingolipid rheostat remodeling in colorectal cancer: global dysregulation, molecular architecture, integrated pathway score, and anatomical variation. (**A**) Differential expression of four principal sphingolipid rheostat-associated genes (SGPP2, CERS6, CERS4, and SGPL1) across normal colorectal tissue (GTEx), colon adenocarcinoma (TCGA COAD), rectal adenocarcinoma (TCGA READ), and pooled colorectal cancer (CRC) samples. Data are presented as median log_2_(TPM + 1) expression values. The magnitude of CRC-associated transcriptional alterations (Δ median expression between pooled CRC and normal tissue) and FDR-adjusted *q* values are shown. SGPP2, CERS6, and SGPL1 were consistently upregulated, whereas CERS4 was markedly downregulated in CRC. (**B**) Transcriptomic profile of 15 rheostat-related genes encompassing sphingosine metabolism, ceramide synthases, and S1P receptor signaling. The heatmap displays log_2_ fold changes relative to normal tissue for colon cancer, rectal cancer, and pooled CRC, demonstrating broadly concordant pathway-level remodeling across tumor sites. Red and blue indicate positive and negative log_2_ fold changes, respectively. (**C**) Sample-level sphingolipid rheostat remodeling score (SRRS) derived from the integrated expression profile of rheostat-associated genes. The left panel shows the shift in SRRS in colon and rectal CRC relative to normal colorectal tissue, whereas the right panel shows the direction and magnitude of CRC-associated dysregulation of individual genes contributing to the score. Blue and red bars indicate negative and positive contributions to the SRRS, respectively. (**D**) Anatomical variation in four selected principal rheostat-associated genes along the cecum-to-rectum axis. Median z-scored expression was assessed across eight anatomical colorectal regions, and associations with ordered anatomical position were evaluated using Spearman correlation. The selected genes did not exhibit a significant common association with anatomical position, indicating relative preservation of the principal rheostat-associated expression pattern across colorectal locations. A separate unbiased analysis of the complete set of 23 evaluable sphingolipid-related genes identified six genes with significant individual associations with anatomical position after FDR correction, as reported in Section 3.11 and Supplementary Figure S1. Green and red indicate genes with increased and decreased expression in CRC, respectively.

Among the principal rheostat-associated genes shown in Figure 7A, SGPP2, CERS6, and SGPL1 were consistently upregulated in CRC, whereas CERS4 showed marked downregulation relative to normal colorectal tissue. These alterations were observed across both colon and rectal cancer datasets and were retained in the pooled CRC analysis.

A broader analysis of 15 rheostat-related genes demonstrated coordinated transcriptional alterations across several branches of sphingolipid metabolism, including sphingosine turnover, ceramide synthases, and S1P receptor signaling (Figure 7B). The overall direction of transcriptional changes was largely concordant between COAD and READ, despite gene-specific differences in effect magnitude.

At the pathway level, the sphingolipid rheostat remodeling score (SRRS) showed a significant shift in both colon and rectal CRC relative to normal colorectal tissue (Figure 7C). SGPP2 and CERS6 were among the dominant positive contributors to the altered transcriptional signature, whereas CERS4, CERS1, and selected S1P receptor components contributed in the opposite direction.

These findings provide complementary transcriptomic evidence that sphingolipid-pathway genes are altered in CRC. However, transcript abundance does not directly measure enzyme activity, metabolic flux, or lipid concentrations; accordingly, the TCGA–GTEx analysis is interpreted as pathway-level molecular context rather than direct validation of the lipidomic findings.

### 3.10. Transcriptomic Variation Along the Colorectal Anatomical Axis

To examine transcriptomic variation across colorectal anatomy, RNA-sequencing profiles from 374 TCGA colorectal tumors with detailed anatomical annotation were analyzed according to an ordered cecum-to-rectum anatomical axis. The cohort included tumors from the cecum (*n* = 78), ascending colon (*n* = 61), hepatic flexure (*n* = 17), transverse colon (*n* = 23), splenic flexure (*n* = 4), descending colon (*n* = 15), sigmoid colon (*n* = 83), and rectum (*n* = 92). One rectosigmoid junction tumor was assigned to the sigmoid–rectal transition within the predefined anatomical ordering.

Principal component analysis of the genome-wide expression matrix showed that the dominant source of transcriptomic variation was not associated with anatomical position. In contrast, PC2 was significantly correlated with position along the ordered cecum-to-rectum axis (Spearman *ρ* = 0.405, *p* < 0.0001).

Genome-wide analysis of 19,885 genes identified 3376 genes (16.98%) whose expression was significantly associated with anatomical position after multiple-testing correction. Of these, 2584 showed increasing expression toward the distal colorectum, whereas 792 showed increasing expression toward the proximal colorectum, corresponding to a 3.26-fold predominance of distal-enriched transcripts. Among the strongest associations, PRAC1 and LY6G6D showed distal enrichment, whereas HOXC6, ARHGAP4, and FOXD1 showed proximal enrichment. Genome-wide anatomical results are provided in Supplementary Table S15.

Functional enrichment analysis of genes associated with anatomical position identified distinct biological programs across the colorectal axis. Distal-enriched genes were associated predominantly with MYC-related programs, ribosome biogenesis, RNA processing, and biosynthetic processes, whereas proximal-enriched genes showed greater representation of interferon signaling, antigen presentation, and T-cell- and natural killer cell-related programs.

Because anatomical subsites were unevenly represented, particularly at the splenic and hepatic flexures and in the descending colon, these findings should be interpreted as population-level associations between gene expression and ordered colorectal position rather than as evidence of uniform or strictly continuous expression changes between adjacent anatomical segments.

### 3.11. Anatomical Variation in Sphingolipid-Pathway Gene Expression

The predefined panel of 23 evaluable sphingolipid-related genes was examined for associations with position along the ordered cecum-to-rectum anatomical axis. Six genes remained significantly associated with anatomical position after FDR correction.

Distal enrichment was observed for SPTLC1 (*ρ* = 0.137; *q* = 0.0308), CERS2 (*ρ* = 0.172; *q* = 0.0047), and CERS4 (*ρ* = 0.208; *q* = 0.0006). In contrast, DEGS2 (*ρ* = −0.285; *q* < 0.0001), SPTLC3 (*ρ* = −0.190; *q* = 0.0017), and SGPP2 (*ρ* = −0.137; *q* = 0.0308) showed proximal enrichment (Supplementary Figure S1 and Table S16).

These anatomical associations involved several components of sphingolipid metabolism, including de novo sphingolipid synthesis (SPTLC1, SPTLC3), ceramide biosynthesis and remodeling (CERS2, CERS4, DEGS2), and S1P metabolism (SGPP2).

This unbiased 23-gene analysis should be distinguished from Figure 7D, which evaluates four biologically selected principal rheostat-associated genes. The selected genes shown in Figure 7D did not exhibit a significant common association with anatomical position, whereas the broader unbiased panel identified six individual genes with significant position-associated expression patterns. Given the unequal representation of anatomical subsites and the modest effect sizes of several correlations, these findings should be interpreted as population-level associations with anatomical position rather than evidence of uniform expression gradients across consecutive colorectal segments.

## 4. Discussion

The present study provides an integrated view of sphingolipid remodeling in colorectal cancer by combining patient-matched tissue lipidomics, systemic oxidative stress profiling, and complementary transcriptomic analyses. Three main observations emerge. First, tumor-associated sphingolipid remodeling involved coordinated changes among pathway components, with S1P-to-ceramide ratios showing a strongly organized covariance structure alongside alterations in individual metabolites. Second, metabolic heterogeneity did not resolve into stable lipidomic subclasses within the present cohort and was compatible with a continuum-like organization along the dominant axes of variation. Third, systemic redox alterations were observed alongside local sphingolipid remodeling, while TCGA–GTEx analyses provided complementary transcriptomic context for dysregulation of sphingolipid-related pathways in CRC. Together, these observations provide the basis for a conceptual CRC Metabolic Continuum framework integrating complementary levels of metabolic organization, while recognizing that its biological and clinical significance requires validation in larger independent cohorts.

A major strength of the study is its patient-matched design. Comparing tumor tissue directly with adjacent non-neoplastic mucosa from the same patient reduces the influence of interindividual differences in genetic background, diet, lifestyle, systemic metabolism, and other sources of biological variability. The resulting Δ(T − N) profiles therefore provide a more direct measure of tumor-associated metabolic remodeling than comparisons between unrelated tumor and control populations and serve as a consistent biological reference for integration with circulating and transcriptomic data.

A central finding was that sphingolipid remodeling involved coordinated relationships among pathway components rather than isolated alterations of individual lipid species. Previous studies have reported changes in selected ceramides and sphingosine-1-phosphate (S1P) in CRC [17,27,28,29], but individual metabolites capture only part of the organization of this highly interconnected pathway. In the present study, S1P-to-ceramide ratios showed a strongly coordinated covariance structure, with a greater concentration of variance along the first principal component in the ratio-derived feature space than in the absolute-lipid feature space. Because these ratios are mathematically derived from their component metabolites and share S1P as a common numerator, this pattern should be interpreted as a description of the internal covariance structure rather than as evidence that ratio-derived variables independently outperform absolute lipid concentrations. Importantly, an altered S1P-to-ceramide ratio cannot by itself distinguish changes driven by enzyme activity from those resulting from altered substrate availability, synthesis, degradation, or turnover of the component metabolites.

This pattern is consistent with the organization of sphingolipid metabolism. Ceramides, sphingosine, and S1P are continuously interconverted through tightly regulated enzymatic reactions and participate in signaling networks controlling proliferation, apoptosis, inflammation, cellular plasticity, and responses to metabolic stress [9,10,11,12,13]. The sphingolipid rheostat is therefore unlikely to operate as a simple balance between two opposing metabolites, but rather as an emergent state of coordinated pathway activity. Our previous experimental work similarly showed that perturbation of individual enzymatic nodes, including SPHK1 and SGPL1, induces remodeling of multiple sphingolipid species rather than isolated changes in single metabolites [27]. The present findings extend this concept to human CRC tissue by demonstrating coordinated variation among multiple components of the sphingolipid network.

PCA, UMAP, t-SNE, and complementary clustering diagnostics did not identify stable lipidomic subclasses within the present cohort, instead showing graded variation across the dominant metabolic space. This pattern is compatible with a continuum-like organization of sphingolipid heterogeneity in CRC rather than clearly separated metabolic phenotypes. However, given the modest cohort size, the absence of detectable stable clusters cannot exclude smaller, overlapping, or weakly separated biologically meaningful subgroups. Similar shifts from categorical classifications toward continuous biological states have emerged in transcriptomic, epigenetic, and microenvironmental studies of cancer, where molecular heterogeneity frequently extends across conventional subtype boundaries [30,31,32,33]. Such an organization, if confirmed in larger independent cohorts, may also help explain why individual sphingolipid biomarkers have shown heterogeneous and partially overlapping associations across clinical studies [34,35,36,37].

Individual ceramide species nevertheless showed distinct patterns of tumor-associated remodeling. C20-Cer exhibited the most consistent tumor-associated depletion and relatively low interindividual variability, whereas S1P-derived variables, particularly S1P-to-ceramide ratios, showed strong descriptive associations with the dominant metabolic axis. These associations should be interpreted within the covariance structure of the multivariate dataset rather than as independent evidence of superior predictive performance. This heterogeneity is consistent with chain-length-specific ceramide biology. Long-chain and very-long-chain ceramides differ in membrane organization, intracellular trafficking, mitochondrial signaling, inflammatory regulation, and susceptibility to enzymatic interconversion [9,10,11,12,13,36,37]. Our previous clinical observations similarly showed preferential remodeling of individual long-chain and very-long-chain ceramide species during CRC progression [14]. These findings support a multidimensional interpretation of the sphingolipid rheostat in which ceramide composition and its relationship with S1P may provide information that is obscured when ceramides are considered only as a total pool.

External lipidomic validation was not available. Nevertheless, increased S1P-associated measures, preferential C20-Cer depletion, and altered S1P-to-ceramide ratios were directionally consistent with previous studies of CRC sphingolipid metabolism and with our independent rectal cancer cohort [15,16,28,29]. These similarities represent biological consistency rather than formal external validation and require confirmation in prospective multicenter lipidomic studies.

A further important observation was the limited correspondence between tumor-local sphingolipid remodeling and circulating profiles. No tissue–serum correlations remained significant after correction for multiple testing, and multiblock integration identified only limited shared variation between the two compartments. This limited tissue–circulation correspondence is consistent with our earlier observation that plasma ceramide concentrations do not directly reflect ceramide abundance in CRC tissue [14]. Circulating sphingolipids are influenced by lipid transport and redistribution, hepatic metabolism, immune activation, platelet function, endothelial signaling, and other systemic processes [14,15,16,38,39,40]. Serum concentrations should therefore not be expected to reproduce tumor tissue profiles directly and may instead capture physiological responses occurring beyond the tumor itself.

Oxidative stress provided a complementary systemic dimension. Redox homeostasis and sphingolipid metabolism are closely interconnected: reactive oxygen species regulate enzymes involved in sphingolipid synthesis, ceramide turnover, and S1P metabolism, while sphingolipid mediators influence mitochondrial function, inflammatory signaling, endothelial homeostasis, antioxidant responses, and intracellular ROS generation [4,9,10,11,12,13]. TAC, TOS, and OSI integrate systemic oxidant burden and antioxidant capacity rather than measuring a single redox pathway, and their pronounced alteration in CRC may reflect the combined effects of inflammation, mitochondrial dysfunction, immune activation, endothelial responses, and metabolic stress [2,4]. Thus, systemic redox status should be considered complementary to, rather than a surrogate for, tumor-local sphingolipid remodeling.

This distinction is also relevant to biomarker interpretation. Oxidative stress markers showed the strongest apparent within-cohort discrimination between patients with CRC and hospitalized non-cancer controls, while sphingolipid-derived metrics characterized tumor-associated metabolic organization [10,15,28,29,41,42]. Combined models achieved the highest apparent within-cohort discriminatory performance, suggesting that redox and sphingolipid variables may contain partly non-overlapping information. However, the control group was not prospectively matched to the CRC cohort for demographic and clinical characteristics, and systemic oxidative-stress markers may be influenced by comorbidities, medication use, inflammation, nutritional status, and other host-related factors. These estimates should therefore not be interpreted as CRC-specific diagnostic or screening performance or as externally validated measures of diagnostic accuracy. More broadly, discriminatory performance and biological informativeness should not be considered interchangeable properties, and future biomarker studies may benefit from integrating systemic markers with pathway-based metabolic measures in appropriately matched and independently validated cohorts.

The transcriptomic analyses provided complementary molecular context for the lipidomic observations through two distinct approaches. TCGA–GTEx comparisons showed altered expression of multiple sphingolipid-pathway components in CRC relative to normal colorectal tissue, while the anatomically resolved TCGA analysis examined molecular variation along the ordered cecum-to-rectum axis. These analyses provide pathway-level transcriptomic context rather than direct validation of individual lipid concentrations, because transcript abundance, enzyme activity, metabolic flux, and tissue lipid levels represent distinct levels of biological regulation.

Genome-wide analysis identified 3376 genes whose expression was significantly associated with position along the colorectal anatomical axis. Distal enrichment involved predominantly proliferative, biosynthetic, and RNA-processing programs, whereas proximal enrichment was more strongly associated with immune signaling and antigen presentation [21,22,23]. Significant associations with anatomical position were also observed for SPTLC1, SPTLC3, CERS2, CERS4, DEGS2, and SGPP2, spanning de novo sphingolipid synthesis, ceramide biosynthesis and remodeling, and S1P metabolism. The involvement of several distinct pathway branches places sphingolipid metabolism within the broader spatial molecular organization of CRC rather than attributing the observed pattern to a single enzymatic component. Given the unequal representation of individual anatomical subsites and the modest effect sizes of several gene-level correlations, these findings should be interpreted as population-level spatial associations rather than uniform expression gradients across consecutive colorectal segments.

Together, the two transcriptomic analyses address distinct aspects of the biological framework. TCGA–GTEx comparisons demonstrate CRC-associated differences in the expression of sphingolipid-pathway genes, whereas the analysis of 374 anatomically annotated tumors identifies associations between gene expression and colorectal anatomical position. Neither analysis constitutes direct external validation of the lipidomic phenotype; rather, they provide complementary transcriptomic context for its biological interpretation. Residual cohort- and source-related effects in the TCGA–GTEx comparison cannot be completely excluded despite uniform Toil processing and should also be considered when interpreting these findings.

The findings also have potential implications for therapeutic studies targeting cancer metabolism. Sphingolipid pathways contain multiple interconnected enzymatic nodes, and perturbation of one component can induce compensatory changes elsewhere in the network [9,10,11,12,13,27]. Therapeutic effects may therefore warrant evaluation at the pathway level, including ceramide composition, S1P metabolism, and redox status, rather than through individual lipid species alone. The interaction between sphingolipid metabolism and oxidative stress may be particularly relevant because both systems regulate mitochondrial function, apoptosis, inflammatory signaling, and cellular adaptation to metabolic stress [4,9,10,11,12,13]. However, the present observational study cannot establish causality or therapeutic responsiveness, and these implications remain hypothesis-generating.

The principal strengths of this study are the patient-matched tissue design, quantitative UHPLC–MS/MS profiling, integration of systemic oxidative stress measurements, and complementary computational analyses spanning metabolic and transcriptomic levels. The biochemical observations were placed in complementary molecular context through TCGA–GTEx comparison of sphingolipid-pathway expression and genome-wide analysis of anatomically resolved TCGA tumors. This design enabled CRC-associated metabolism to be investigated across local, systemic, and molecular scales while reducing reliance on any single analytical modality.

Several limitations should be considered. First, the lipidomic component was based on a relatively small, single-center cohort without an independent external lipidomic cohort. Although the paired design improves the precision of within-patient comparisons, it does not eliminate limitations related to sample size, population specificity, or external generalizability. Second, the cross-sectional design does not establish temporal or causal relationships among sphingolipid remodeling, oxidative stress, and tumor progression. Longitudinal sampling will be required to determine whether the identified metabolic organization changes with tumor burden, disease progression, or treatment.

Third, TAC, TOS, and OSI provide integrated measures of systemic redox status rather than pathway-specific measurements and cannot identify the cellular source of altered oxidant or antioxidant activity. Similarly, circulating sphingolipids are influenced by multiple tissues and physiological compartments and cannot be regarded as direct surrogates of tumor lipid metabolism [14,15,16,38,39,40]. In addition, the non-cancer control group was recruited among individuals hospitalized for non-neoplastic conditions, but detailed matching for demographic characteristics, comorbidities, medication use, and other factors potentially affecting systemic redox status was not available. This limitation should be considered when interpreting between-group differences in circulating redox markers and their apparent discriminatory performance.

Fourth, biomarker analyses were exploratory and performed within the discovery cohort. The high apparent discriminatory performance of oxidative stress markers and combined models therefore requires independent validation before any diagnostic interpretation.

Fifth, transcriptomic analyses provide complementary molecular context rather than direct validation of lipidomic measurements. Transcript abundance does not necessarily correspond to enzyme activity, metabolic flux, or lipid concentration. Moreover, although TCGA and GTEx expression data were obtained from the uniformly processed Toil dataset, residual cohort- and source-related effects cannot be completely excluded. The anatomical analysis was based on bulk RNA sequencing of tumors classified by colorectal location and should not be interpreted as spatial transcriptomics at cellular or tissue-microdomain resolution. In addition, the unequal representation of individual anatomical subsites limits the interpretation of position-associated expression patterns as uniform gradients across consecutive colorectal segments. Integration with spatial metabolomics, true spatial transcriptomics, single-cell profiling, proteomics, and functional experiments will be required to resolve the cellular mechanisms underlying these relationships.

Finally, the CRC Metabolic Continuum should be regarded as a conceptual framework rather than a formally validated predictive model. The absence of stable clusters in the present cohort is compatible with, but does not establish, a continuous biological organization of sphingolipid remodeling. Its generalizability and mechanistic basis require independent testing in larger, prospectively collected cohorts.

## 5. Conclusions

This study supports a systems-level view of metabolic remodeling in colorectal cancer. Patient-matched tissue analyses showed coordinated alterations across the sphingolipid pathway, with S1P-to-ceramide relationships contributing prominently to the observed covariance structure. Metabolic heterogeneity did not resolve into stable lipidomic subclasses within the present cohort and was compatible with a continuum-like organization along the dominant axes of variation. Circulating profiles revealed a distinct systemic dimension characterized by pronounced redox alterations, with limited direct correspondence to tumor-local sphingolipid remodeling.

TCGA–GTEx comparisons and anatomically resolved TCGA analyses provided complementary transcriptomic context by demonstrating CRC-associated alterations in sphingolipid-pathway gene expression and associations between gene expression and colorectal anatomical position. Together, these findings provide the basis for a conceptual CRC Metabolic Continuum integrating local sphingolipid remodeling, systemic redox alterations, and anatomical molecular organization. This framework remains hypothesis-generating and requires validation in larger independent cohorts before its potential biomarker or therapeutic relevance can be established.

## Data Availability

The data generated during the experimental component of this study are available from the corresponding author upon reasonable request. Patient-level experimental source data are not publicly deposited because the original research files contain confidential clinical information. Publicly available transcriptomic datasets from The Cancer Genome Atlas (TCGA) and the Genotype-Tissue Expression (GTEx) project were used for independent transcriptomic analyses. These datasets are available through their respective public repositories. The consolidated and documented analysis code corresponding to the final computational analyses reported in this study is publicly available in the CRC-Sphingolipid-Continuum GitHub repository (https://github.com/adromax68/CRC-Sphingolipid-Continuum; accessed on 8 September 2026) and has been permanently archived in Zenodo (version v1.0.4; DOI: 10.5281/zenodo.22162846; accessed on 8 September 2026). Artificial intelligence tools were used for language polishing of the manuscript and to assist in the preparation of the graphical abstract. All AI-assisted content, including the graphical elements, was subsequently reviewed and verified by the authors for scientific accuracy. The authors take full responsibility for the final content of the manuscript and graphical abstract.

## Supplementary Materials

The following supporting information can be downloaded at https://www.mdpi.com/article/10.3390/antiox15091136/s1. Figure S1: Anatomical variation in sphingolipid-pathway gene expression along the cecum-to-rectum axis; Table S1: Clinical and pathological characteristics of the colorectal cancer cohort; Table S2: Paired tumor–normal differences in sphingolipid variables; Table S3: Top paired alterations ranked by effect size; Table S4: Principal component summary; Table S5: Dominant PCA loadings; Table S6: Cluster-structure diagnostics; Table S7: Comparison of ratio-derived and absolute-lipid feature spaces; Table S8: Variance decomposition of key rheostat ratios; Table S9: Reduced-panel covariance retention analysis; Table S10: Serum CRC-versus-control differences; Table S11: Top serum ROC markers; Table S12: Decision-curve summary and clinical utility ranking; Table S13: Top-ranked tissue–serum correlations; Table S14: Multicollinearity diagnostics for ratio variables; Table S15: Genome-wide transcriptomic associations with anatomical position along the cecum-to-rectum axis in colorectal cancer; Table S16: Associations of sphingolipid-pathway gene expression with anatomical position along the cecum-to-rectum axis in the TCGA colorectal cancer cohort.

## Abbreviations

AJCC, American Joint Committee on Cancer; AUC, Area Under the Curve; BIC, Bayesian Information Criterion; Cer, Ceramide; CRC, Colorectal Cancer; CV, Coefficient of Variation; DCA, Decision Curve Analysis; FDR, False Discovery Rate; IQR, Interquartile Range; LC, Long Chain; NLDR, Nonlinear Dimensionality Reduction; OSI, Oxidative Stress Index; PCA, Principal Component Analysis; PLS, Partial Least Squares; ROC, Receiver Operating Characteristic; S1P, Sphingosine-1-Phosphate; SD, Standard Deviation; TAC, Total Antioxidant Capacity; TNM, Tumor, Node, Metastasis; TOS, Total Oxidant Status; UHPLC-MS/MS, Ultra-High-Performance Liquid Chromatography Tandem Mass Spectrometry; UMAP, Uniform Manifold Approximation and Projection; VLC, Very-Long-Chain; t-SNE, t-distributed Stochastic Neighbor Embedding.
