# Supplementary material for: Sphingolipid Remodeling in Colorectal Cancer Reveals a Continuum-like Metabolic Organization
Source: Antioxidants (Basel). 2026 Sep 8;15(9):1136. doi: 10.3390/antiox15091136 (PMC13603189; doi:10.3390/antiox15091136)
Supplement: Supplementary file 1 [file antioxidants-15-01136-s001.zip › antioxidants-4507587-supplementary.pdf]

Supplementary Materials
Sphingolipid Remodeling in Colorectal Cancer Reveals a Continuum-Like Metabolic Organization

Table S1. Clinical and pathological characteristics of the colorectal cancer cohort. Summary of the 40 patients included in paired analyses of tumor tissue, matched adjacent histologically normal mucosa, and serum. Continuous variables are presented as mean, standard deviation (SD), median, and interquartile range (Q1–Q3), whereas categorical variables are shown as counts. Tumors were classified according to histologic grade and AJCC TNM staging system. Abbreviations: CRC, colorectal cancer; SD, standard deviation; Q1, first quartile; Q3, third quartile; TNM, tumor-node-metastasis; T, primary tumor category; N, nodal category; M, distant metastasis category.

Metric	Value
CRC cohort size	40
Sex: male, n	22
Sex: female, n	18
Age, mean	71.825
Age, SD	9.984
Age, median	72.500
Age Q1	66.000
Age Q3	78.250
Grade G2, n	36
Grade G3, n	3
Grade G4, n	1
TNM I, n	2
TNM II, n	16
TNM III, n	17
TNM IV, n	5
T=1, n	1
T=2, n	11
T=3, n	25
T=4, n	3
N=0, n	19
N=1, n	14
N=2, n	7
M=0, n	35
M=1, n	5

Table S2. Paired tumor–normal differences in sphingolipid variables. Full paired comparison table ranked by false-discovery-rate significance (lowest FDR at top). Variables with the strongest and most statistically robust paired tumor–normal differences are presented first, followed by weaker or non-significant findings. Values are shown as medians with interquartile ranges (IQR). Tumor-associated remodeling was assessed using the Wilcoxon signed-rank test for paired samples, with Benjamini–Hochberg false-discovery-rate (FDR) correction for multiple comparisons. Median Δ(T−N) indicates the median within-patient paired difference between tumor tissue (T) and matched adjacent non-neoplastic mucosa (N). Positive values indicate higher levels in tumor tissue, whereas negative values indicate lower levels. The 95% CI for Δ represents the 95% confidence interval for the paired median difference. The Wilcoxon rank-biserial correlation coefficient r_{rb} is reported as a non-parametric effect-size measure for paired comparisons and was calculated from the positive and negative signed-rank sums as $r_{rb} = (W_{+}-W_{-})/(W_{+}+W_{-})$. Positive r_{rb} values indicate higher levels in tumor tissue relative to matched adjacent non-neoplastic mucosa, whereas negative r_{rb} values indicate lower levels; larger absolute values indicate stronger paired effects. "Direction in tumor" summarizes whether the analyte was higher or lower in tumor tissue relative to matched adjacent non-neoplastic mucosa. Individual sphingolipid concentrations are expressed as pmol/mg tissue; sphingolipid ratios are dimensionless. Abbreviations: Cer, ceramide; Tot-Cer, total ceramides; Sph, sphingosine; S1P, sphingosine-1-phosphate; SPA, sphinganine; C14-, C16-, C18:1-, C18-, C20-, C22-, C24:1-, and C24-Cer denote ceramide species according to acyl-chain length and saturation.

Analyte	Tumor median (IQR)	Normal median (IQR)	Median Δ(T−N)	95% CI for Δ	FDR	Wilcoxon r_{rb}	Direction in tumor
C20-Cer	0.9140 (0.6759–1.274)	1.708 (1.457–2.024)	-0.8485	-0.9868 to -0.5806	<0.0001	−1.0000	Lower
Sph	4.592 (4.215–6.167)	2.208 (1.554–2.781)	2.390	1.876 to 3.829	<0.0001	0.9951	Higher
S1P/C20-Cer	0.0514 (0.0284–0.0709)	0.0170 (0.0092–0.0237)	0.0302	0.0190 to 0.0417	<0.0001	0.9366	Higher
S1P/C18-Cer	0.0180 (0.0116–0.0259)	0.0093 (0.0058–0.0136)	0.0072	0.0032 to 0.0130	<0.0001	0.7756	Higher
S1P	0.0454 (0.0332–0.0605)	0.0292 (0.0152–0.0399)	0.0147	0.0067 to 0.0272	0.0001	0.7098	Higher
C24-Cer	6.628 (4.933–7.930)	4.920 (4.459–5.760)	1.554	0.7716 to 2.658	0.0021	0.6024	Higher
S1P/C24-1-Cer	0.0026 (0.0018–0.0041)	0.0017 (0.00087–0.0022)	0.0010	0.00008 to 0.0018	0.0025	0.5878	Higher
S1P/Tot-Cer	0.000326 (0.000215–0.000478)	0.000198 (0.000123–0.000298)	0.000099	0.000026 to 0.000164	0.0042	0.5585	Higher

Analyte	Tumor median (IQR)	Normal median (IQR)	Median Δ(T−N)	95% CI for Δ	FDR	Wilcoxon r_{rb}	Direction in tumor
S1P/C16-Cer	0.000416 (0.000263–0.000635)	0.000261 (0.000154–0.000394)	0.000156	0.000029 to 0.000226	0.0043	0.5512	Higher
S1P/C22-Cer	0.0160 (0.0110–0.0236)	0.0110 (0.0057–0.0155)	0.0030	0.0011 to 0.0095	0.0053	0.5366	Higher
S1P/C18-1-Cer	0.1195 (0.0674–0.1856)	0.0749 (0.0405–0.1343)	0.0396	0.0077 to 0.0739	0.0145	0.4780	Higher
C14-Cer	1.282 (1.034–1.456)	0.9740 (0.7328–1.304)	0.2307	0.0747 to 0.4749	0.0210	0.4512	Higher
C18-Cer	2.781 (1.619–3.280)	3.034 (2.267–3.650)	-0.3105	-0.7965 to -0.0549	0.0335	−0.4171	Lower
S1P/C24-Cer	0.0083 (0.0045–0.0112)	0.0053 (0.0031–0.0073)	0.0012	-0.00049 to 0.0037	0.0876	0.3439	Higher
S1P/C14-Cer	0.0374 (0.0238–0.0480)	0.0268 (0.0138–0.0440)	0.0057	-0.0016 to 0.0183	0.1078	0.3220	Higher
SPA	0.8242 (0.6278–1.310)	0.7918 (0.5595–1.083)	0.0637	-0.0898 to 0.2849	0.1416	0.2829	Higher
C16-Cer	113.47 (80.749–153.24)	104.65 (77.972–121.81)	18.605	-8.926 to 34.502	0.1416	0.2902	Higher
Tot-Cer	142.92 (112.26–182.11)	138.29 (109.29–155.38)	16.789	-12.241 to 37.731	0.1416	0.2854	Higher
C22-Cer	3.026 (2.287–3.451)	2.734 (2.057–3.126)	0.1108	-0.0889 to 0.4104	0.2447	0.2244	Higher
C18-1-Cer	0.3686 (0.2877–0.4853)	0.3522 (0.2407–0.4549)	0.0301	-0.0513 to 0.0668	0.5908	0.1073	Higher
C24-1-Cer	17.205 (14.686–20.195)	17.531 (15.204–19.847)	-0.6154	-1.746 to 1.236	0.6850	−0.0756	Lower

Table S3. Top paired alterations ranked by effect size. The strongest tumor-associated sphingolipid changes identified across the paired colorectal cancer cohort are shown, ranked according to the absolute value of the Wilcoxon rank-biserial correlation coefficient (|rrb||r_{rb}|). The Wilcoxon rank-biserial correlation coefficient is a non-parametric effect-size measure for paired comparisons and was calculated from the positive and negative signed-rank sums as $r_{rb} = (W_{+}−W_{-})/(W_{+}+W_{-})$. Larger absolute values indicate stronger paired tumor–normal effects. Positive r_{rb} values indicate higher levels in tumor tissue relative to matched adjacent non-neoplastic mucosa, whereas negative r_{rb} values indicate lower levels. FDR values represent Benjamini–Hochberg FDR-adjusted P-values accounting for multiple comparisons. "Direction" summarizes whether each analyte was increased or decreased in tumor tissue. Only the strongest paired alterations are shown. Individual sphingolipid concentrations are expressed as pmol/mg tissue; sphingolipid ratios are dimensionless. Abbreviations: Cer, ceramide; Tot-Cer, total ceramides; Sph, sphingosine; S1P, sphingosine-1-phosphate; C14-, C16-, C18:1-, C18-, C20-, C22-, C24:1-, and C24-Cer denote ceramide species according to acyl-chain length and saturation.

Rank	Analyte	Wilcoxon r_{rb}	FDR	Direction
1	C20-Cer	−1.0000	<0.0001	Lower in tumor
2	Sph	0.9951	<0.0001	Higher in tumor
3	S1P/C20-Cer	0.9366	<0.0001	Higher in tumor
4	S1P/C18-Cer	0.7756	<0.0001	Higher in tumor
5	S1P	0.7098	0.0001	Higher in tumor
6	C24-Cer	0.6024	0.0021	Higher in tumor
7	S1P/C24-1-Cer	0.5878	0.0025	Higher in tumor
8	S1P/Tot-Cer	0.5585	0.0042	Higher in tumor
9	S1P/C16-Cer	0.5512	0.0043	Higher in tumor
10	S1P/C22-Cer	0.5366	0.0053	Higher in tumor
11	S1P/C18-1-Cer	0.4780	0.0145	Higher in tumor
12	C14-Cer	0.4512	0.0210	Higher in tumor

Table S4. Principal component summary. Low-dimensional summary of the paired tumor–normal remodeling matrix derived from principal component analysis (PCA). PCA was applied to the paired within-patient remodeling dataset to identify the dominant latent axes explaining covariance among sphingolipid variables. Principal component 1 (PC1) accounted for 37.7% of the total variance and was characterized predominantly by sphingosine-1-phosphate (S1P) and related S1P-to-ceramide ratio indices. Principal component 2 (PC2) accounted for 15.0% of the total variance and was associated mainly with ceramide composition and chain-length-dependent variation within the ceramide pool. Together, PC1 and PC2 explained 52.8% of the total variance. "Variance explained" indicates the proportion of total dataset variance accounted for by each component. Abbreviations: PCA, principal component analysis; PC1, first principal component; PC2, second principal component; S1P, sphingosine-1-phosphate.

Component	Variance explained	Interpretation
PC1	37.7%	Dominant rheostat-centered metabolic axis
PC2	15.0%	Secondary ceramide-composition axis
PC1+PC2	52.8%	Low-dimensional structure captured by first two components

Table S5. Dominant PCA loadings. Variables with the strongest contributions to the first two principal components identified in the principal component analysis (PCA) of the paired tumor–normal remodeling matrix. Loadings quantify the relative contribution and directional alignment of each variable with a given component. Higher absolute loading values indicate stronger contributions to component structure. Positive and negative loadings indicate opposite directions of association with the corresponding component and should be interpreted relative to the overall loading pattern. In this dataset, PC1 was characterized predominantly by positive loadings of sphingosine-1-phosphate (S1P) and multiple S1P-to-ceramide ratio variables, consistent with an S1P-associated remodeling axis. PC2 was characterized mainly by loadings related to total ceramides and selected ceramide species, consistent with variation in ceramide composition and chain-length distribution. Loadings represent the contribution of variables to multivariate covariance structure and should not be interpreted as effect sizes or measures of statistical significance. Abbreviations: PCA, principal component analysis; PC1, first principal component; PC2, second principal component; S1P, sphingosine-1-phosphate; Cer, ceramide; Tot-Cer, total ceramides; SPA, sphinganine; C14-, C16-, C18-, C20-, C22-, C24-, and C24:1-Cer denote ceramide species according to acyl-chain length and saturation.

Variable	PC1 loading	PC2 loading
S1P	0.3318	-0.1330
S1P/Tot-Cer	0.3286	0.1516
S1P/C24-1-Cer	0.3203	-0.1056
S1P/C24-Cer	0.3127	-0.0288
S1P/C16-Cer	0.3073	0.2007
S1P/C18-Cer	0.2933	0.1372
S1P/C22-Cer	0.2858	-0.2099
S1P/C18-1-Cer	0.2858	-0.1313
S1P/C20-Cer	0.2727	-0.1867
S1P/C14-Cer	0.2341	-0.0043
Tot-Cer	-0.0824	-0.5099
C16-Cer	-0.0574	-0.5034
C18-Cer	0.0143	-0.4386
C14-Cer	0.0778	-0.1544
SPA	-0.1546	-0.1388

Table S6. Cluster-Structure Diagnostics. Multiple complementary unsupervised diagnostics were applied to assess whether the multivariable tumor-associated sphingolipid remodeling data supported robust discrete subgroup structure. Silhouette analysis was evaluated across candidate partitions from k = 2 to 6, and the gap statistic was assessed for k = 1 to 6 using 20 uniformly generated reference datasets, as described in the Methods. The table summarizes the principal candidate solutions relevant to interpretation. Uniformly low silhouette values indicated weak cluster separation, while the gap statistic showed no clear optimum. Gaussian mixture modeling based on the Bayesian information criterion favored a single-component solution. The Hopkins statistic was modestly above 0.5, indicating some non-random internal structure but not strong evidence of a clearly clusterable architecture. Collectively, these complementary analyses provided limited support for robust discrete sphingolipidomic subgroups in this cohort. Given the modest sample size, the absence of detectable stable clusters should not be interpreted as proof of a true biological continuum or as evidence that biologically meaningful, smaller, overlapping, or weakly separated subgroups do not exist. Abbreviations: BIC, Bayesian information criterion; CI, confidence interval; k, number of tested clusters.

Method	Configuration	Result	Interpretation
Silhouette	k=2	0.195	Weak separation
Silhouette	k=3	0.110	Weak separation
Silhouette	k=4	0.108	Weak separation
Silhouette	k=5	0.109	Weak separation
Gap statistic	k=2–4	0.42–0.46	No clear optimum
Gaussian mixture BIC	1 component	-412.6	Single-component solution favored
Hopkins statistic	overall	0.596 (95% CI 0.569–0.620)	Limited evidence of cluster tendency

Table S7. Comparison of ratio-derived and absolute-lipid feature spaces. Principal component analysis (PCA) was performed separately for absolute lipid concentrations, S1P-to-ceramide ratio-derived variables, and the complete 21-variable feature set. The proportion of variance explained by PC1 and PC2 therefore describes the concentration of covariance within each independently analyzed feature space and should not be interpreted as the proportion of a common principal component predicted by either feature class. The ratio-derived feature space showed a more concentrated dominant covariance structure, with PC1 accounting for 69.5% of within-block variance compared with 22.6% in the absolute-lipid feature space and 37.7% in the complete feature space. Because the ratio variables are mathematically derived from their component metabolites and share S1P as a common numerator, these comparisons are descriptive and do not constitute independent evidence that ratio-derived variables outperform absolute lipid concentrations. Abbreviations: PCA, principal component analysis; PC1, first principal component; PC2, second principal component; S1P, sphingosine-1-phosphate.

Feature space	Number of variables	PC1 variance explained	PC2 variance explained
Absolute-only	12	22.6%	16.6%
Ratio-only	9	69.5%	9.5%
Full feature space	21	37.7%	15.0%

Table S8. Variance decomposition of key rheostat ratios. For selected sphingosine-1-phosphate (S1P)-to-ceramide ratios evaluated on the paired log10 tumor–normal scale, total variance was algebraically partitioned into contributions from the

numerator term, denominator term, and covariance term. Because log-transformed ratios can be expressed as differences [Δlog10(S1P) − Δlog10(Cer)], their variance can be decomposed according to the identity Var(A − B) = Var(A) + Var(B) − 2Cov(A,B). "Total variance" represents the observed variance of the paired log-ratio shift across patients. "Numerator variance" reflects the variance of paired changes in S1P, whereas "Denominator variance" reflects the variance of paired changes in the corresponding ceramide denominator. "Covariance contribution (−2Cov)" represents the algebraic contribution of covariance between numerator and denominator changes to the total variance. Negative covariance between numerator and denominator changes therefore produces a positive −2Cov contribution and increases the variance of their difference, whereas positive covariance produces a negative −2Cov contribution and reduces it. Percentage columns express each algebraic component as a proportion of the total variance; accordingly, individual contributions may exceed 100% or be negative, while the three components sum to 100% apart from rounding. In this cohort, between-patient variability in S1P changes accounted for the largest component of variance in the selected rheostat ratio shifts, whereas variability in the corresponding ceramide denominators generally contributed less. This decomposition characterizes inter-individual variability in tumor–normal ratio shifts and should not be interpreted as establishing the relative contribution of numerator and denominator changes to the mean tumor–normal shift or as evidence of a causal mechanism. Abbreviations: S1P, sphingosine-1-phosphate; Cer, ceramide; Tot-Cer, total ceramides; Cov, covariance.

Ratio	Total variance	Numerator variance	Denominator variance	Covariance contribution (−2Cov)	Numerator %	Denominator %	Covariance contribution %
Δlog10(S1P/Tot-Cer)	0.142851	0.110208	0.022521	+0.010121	77.1	15.8	7.1
Δlog10(S1P/C18-Cer)	0.114020	0.110208	0.032090	−0.028278	96.7	28.1	-24.8
Δlog10(S1P/C20-Cer)	0.155019	0.110208	0.029196	+0.015616	71.1	18.8	10.1

Table S9. Reduced-Panel Covariance Retention Analysis. Assessment of whether a reduced three-ratio panel preserves the dominant interaction-based structure captured by the full nine-ratio sphingolipid panel. Principal component analysis (PCA) was performed separately for the full and reduced feature sets. "PC1 variance explained" denotes the proportion of within-panel variance captured by the first principal component. The reduced three-ratio panel showed a more concentrated low-dimensional structure than the full ratio panel, with PC1 accounting for 75.93% versus 69.51% of within-panel variance. Direct comparison of the independently derived PC1 scores showed high concordance between the reduced and full panels (R^2 = 0.9351). These findings indicate that the reduced panel retained much of the dominant covariance structure of the full ratio feature space. Abbreviations: PCA, principal component analysis; PC1, first principal component; R^2, coefficient of determination.

Model / comparison	PC1 variance explained	PC1 concordance R^2
Full ratio panel (9 ratios)	69.51%	Reference
Minimal ratio panel (3 ratios)	75.93%	—
Minimal-panel PC1 vs full-panel PC1	—	0.9351

Table S10. Serum CRC-versus-control differences. Univariate comparison of circulating sphingolipid and oxidative-stress variables between patients with colorectal cancer (CRC) and non-cancer serum controls. Continuous variables are presented as medians with interquartile ranges (IQR). "Difference in medians (CRC − control)" represents the difference between the CRC and control group medians, with positive values indicating higher levels in CRC and negative values indicating lower levels in CRC. Group comparisons were performed using the Mann–Whitney U test. False discovery rate (FDR) values represent Benjamini–Hochberg-adjusted *p* values correcting for multiple comparisons across the evaluated serum markers. Rank-biserial correlation (*r*) is reported as a non-parametric effect-size measure describing the magnitude and direction of group separation; positive values indicate higher values in CRC, whereas negative values indicate lower values in CRC, with larger absolute values indicating stronger separation. "Direction" summarizes the direction of the between-group difference. Oxidative-stress markers showed large between-group effect sizes, with lower total antioxidant capacity (TAC) and higher oxidative stress index (OSI) and total oxidant status (TOS) in CRC. Several S1P-to-ceramide ratios were also higher in CRC, whereas selected ceramide species were lower. Abbreviations: CRC, colorectal cancer; IQR, interquartile range; FDR, false discovery rate; TAC, total antioxidant capacity; OSI, oxidative stress index; TOS, total oxidant status; S1P, sphingosine-1-phosphate; Sph, sphingosine; SPA, sphinganine; Cer, ceramide; Tot-Cer, total ceramides; C14-, C16-, C18-, C20-, C22-, C24-, and C24:1-Cer denote ceramide species according to acyl-chain length and saturation.

Marker	CRC median (IQR)	Control median (IQR)	Difference in medians (CRC−control)	FDR	Rank-biserial R	Direction
TAC (μmol/L)	705.27 (615.61–1026.52)	1527.13 (960.23–1845.18)	−821.86	<0.0001	−0.85	Lower in CRC
OSI	1.703 (1.448–2.104)	0.576 (0.460–0.772)	1.127	<0.0001	0.92	Higher in CRC
TOS (μmol/L)	13.144 (11.249–14.936)	7.591 (6.978–9.323)	5.553	<0.0001	0.87	Higher in CRC
S1P/C14	23.271 (17.733–28.039)	15.860 (13.408–17.436)	7.411	<0.0001	0.70	Higher in CRC
S1P/C22	0.569 (0.390–0.673)	0.373 (0.308–0.437)	0.196	0.0002	0.63	Higher in CRC
C14-Cer	16.614 (14.188–18.632)	20.236 (17.485–25.049)	−3.621	0.0004	−0.59	Lower in CRC
C22-Cer	736.59	869.85	−133.26	0.0008	−0.56	Lower in CRC

	(622.61–844.15)	(768.44–958.19)				
S1P/C20	9.459 (7.149–12.120)	6.789 (5.645–7.837)	2.671	0.0028	0.50	Higher in CRC
Sph	9.617 (7.462–11.399)	13.043 (10.702–14.214)	−3.426	0.0028	−0.50	Lower in CRC
S1P/T-Cer	0.0787 (0.0602–0.0927)	0.0623 (0.0547–0.0684)	0.0163	0.0067	0.45	Higher in CRC
S1P	385.60 (311.12–459.62)	314.30 (288.20–328.48)	71.30	0.0067	0.45	Higher in CRC
S1P/C24-1	0.324 (0.266–0.412)	0.253 (0.203–0.312)	0.0705	0.0085	0.43	Higher in CRC
SPA	4.564 (4.001–5.367)	5.648 (4.817–7.807)	−1.084	0.0085	−0.43	Lower in CRC
C16-Cer	470.52 (415.78–519.40)	403.91 (354.28–456.98)	66.61	0.0092	0.43	Higher in CRC
S1P/C18	7.765 (6.268–10.490)	5.685 (5.204–8.013)	2.079	0.0096	0.42	Higher in CRC
S1P/C18-1	58.849 (42.876–78.110)	44.855 (35.250–59.521)	13.99	0.0224	0.37	Higher in CRC
S1P/C24	0.149 (0.126–0.182)	0.125 (0.109–0.143)	0.0232	0.0447	0.33	Higher in CRC
C20-Cer	43.043 (33.554–53.319)	47.787 (39.956–55.913)	−4.744	0.1362	−0.25	Lower in CRC
C18-Cer	46.178 (40.251–55.289)	52.743 (43.009–60.000)	−6.565	0.4376	−0.14	Lower in CRC
S1P/C16	0.861 (0.659–1.070)	0.770 (0.667–0.952)	0.0912	0.4376	0.14	Higher in CRC
C24-Cer	2606.85 (2217.45–2975.91)	2508.74 (2297.03–2628.00)	98.11	0.4613	0.13	Higher in CRC
C18-1-Cer	6.767 (5.594–7.433)	6.572 (5.950–8.615)	0.195	0.5934	−0.09	Higher in CRC
C24-1-Cer	1230.77 (1019.56–1365.01)	1202.01 (1108.08–1302.98)	28.76	0.7917	−0.04	Higher in CRC
Tot-Cer	5171.67 (4779.02–5503.57)	5155.17 (4862.82–5568.84)	16.51	0.7917	−0.05	Higher in CRC

Table S11. Top serum ROC markers. Univariate receiver operating characteristic (ROC) performance of circulating biomarkers for distinguishing patients with colorectal cancer (CRC) from non-cancer serum controls, ranked by area under the ROC curve (AUC). ROC analysis was performed separately for each serum marker. AUC values range from 0.50 (no discrimination) to 1.00 (perfect discrimination), with higher values indicating greater separation between CRC and control groups. “Disease-associated direction” indicates whether higher or lower marker values were associated with CRC. Prior to cutoff optimization, markers were oriented so that the disease-associated direction corresponded to the positive test state. “Youden cutoff” represents the threshold maximizing the Youden index (sensitivity + specificity − 1) within the analyzed cohort. Sensitivity denotes the proportion of CRC cases correctly classified, whereas specificity denotes the proportion of controls correctly classified as non-cancer. Oxidative-stress markers showed the highest univariate AUCs, led by oxidative stress index (OSI), total oxidant status (TOS), and total antioxidant capacity (TAC). Among the top-ranked lipid-derived variables, S1P-to-ceramide ratios showed higher AUCs than the individual sphingolipid species included in this table. These ROC estimates represent internal cohort performance and should not be interpreted as externally validated diagnostic performance or clinically validated thresholds. Abbreviations: ROC, receiver operating characteristic; AUC, area under the curve; CRC, colorectal cancer; TAC, total antioxidant capacity; OSI, oxidative stress index; TOS, total oxidant status; S1P, sphingosine-1-phosphate; Sph, sphingosine; Cer, ceramide.

Rank	Marker	AUC	Disease-associated direction	Youden cutoff	Sensitivity	Specificity
1	OSI	0.960	Higher in CRC	1.13	0.875	0.957
2	TOS (μmol/L)	0.936	Higher in CRC	10.81	0.875	0.913
3	TAC (μmol/L)	0.900	Lower in CRC	1123.35	0.875	0.913
4	S1P/C14	0.850	Higher in CRC	19.59	0.725	0.957
5	S1P/C22	0.815	Higher in CRC	0.493	0.650	0.957
6	C14-Cer	0.795	Lower in CRC	18.92	0.800	0.652
7	C22-Cer	0.782	Lower in CRC	918.10	0.950	0.478
8	Sph	0.752	Lower in CRC	12.12	0.900	0.565
9	S1P/C20	0.750	Higher in CRC	8.08	0.650	0.826
10	S1P	0.726	Higher in CRC	352.23	0.675	0.826

Table S12. Decision-curve summary and clinical utility ranking. Serum markers were ranked according to mean net benefit estimated by decision-curve analysis (DCA) across threshold probabilities ranging from 0.10 to 0.50. DCA evaluates the potential value of marker-guided classification by balancing true-positive and false-positive classifications across decision thresholds and comparing marker-based strategies with default strategies such as treating all individuals or treating none. This table provides a concise summary of the decision-curve results and does not replace inspection of the full decision curves. “Rank” indicates the ordering according to mean net benefit across the evaluated threshold range. “Block” classifies biomarkers as oxidative-stress markers, lipid-ratio markers, or individual lipid species. “AUC” denotes the area under the receiver operating characteristic curve

and is provided as a conventional measure of discrimination. "Mean net benefit" represents the average net benefit across threshold probabilities of 0.10–0.50, whereas "Peak net benefit" indicates the highest net benefit observed within this interval. "Direction" indicates whether higher or lower marker values were associated with colorectal cancer (CRC). Oxidative-stress markers showed the highest mean net benefit across the evaluated threshold range, led by oxidative stress index (OSI), total oxidant status (TOS), and total antioxidant capacity (TAC). Among lipid-derived variables, both S1P-to-ceramide ratios and individual sphingolipid species showed positive net benefit within the evaluated threshold range. These estimates describe internal cohort performance and require external validation before any inference regarding clinical utility or implementation. Abbreviations: DCA, decision-curve analysis; AUC, area under the curve; CRC, colorectal cancer; TAC, total antioxidant capacity; OSI, oxidative stress index; TOS, total oxidant status; S1P, sphingosine-1-phosphate; Sph, sphingosine; SPA, sphinganine; Cer, ceramide.

Rank	Marker	Block	AUC	Mean net benefit	Peak net benefit	Direction
1	OSI	oxidative	0.960	0.556	0.594	Higher in CRC
2	TOS (µmol/L)	oxidative	0.936	0.515	0.585	Higher in CRC
3	TAC (µmol/L)	oxidative	0.900	0.505	0.572	Lower in CRC
4	S1P/C14	lipid_ratio	0.850	0.484	0.560	Higher in CRC
5	C14-Cer	individual_lipid	0.795	0.482	0.548	Lower in CRC
6	Sph	individual_lipid	0.752	0.472	0.535	Lower in CRC
7	C22-Cer	individual_lipid	0.782	0.466	0.532	Lower in CRC
8	S1P/C20	lipid_ratio	0.750	0.464	0.528	Higher in CRC
9	S1P/C22	lipid_ratio	0.815	0.460	0.540	Higher in CRC
10	SPA	individual_lipid	0.717	0.458	0.520	Lower in CRC

Table S13. Top-ranked tissue–serum correlations. Highest-ranking cross-compartment Spearman correlations between paired tissue-remodeling variables and matched serum biomarkers. Tissue variables were expressed as within-patient paired differences between tumor and adjacent normal mucosa [Δ(T−N)], representing tumor-associated local remodeling on the log-transformed scale. Serum variables were measured in matched preoperative blood samples and included circulating sphingolipid and oxidative-stress markers. Spearman's rank correlation coefficient (ρ) was used to assess monotonic associations between local tissue shifts and systemic circulating signals without assuming normality. Positive values indicate that larger tumor-associated increases (or smaller decreases) in tissue variables were associated with higher serum marker levels, whereas negative values indicate inverse relationships. Only the strongest observed correlations are shown. False discovery rate (FDR) values were calculated using the Benjamini–Hochberg procedure across the full matrix of tissue–serum comparisons. Although several moderate nominal correlations were observed, none remained statistically significant after correction for multiple testing, indicating limited detectable tissue–serum correspondence in this cohort. Abbreviations: Δ(T−N), paired tumor-minus-normal difference; ρ, Spearman rank correlation coefficient; FDR, false discovery rate; S1P, sphingosine-1-phosphate; Sph, sphingosine; Cer, ceramide; Tot-Cer, total ceramides; TOS, total oxidant status.

Tissue variable	Serum variable	Spearman ρ	FDR
Δ-C22-Cer	S1P/C20	-0.4066	0.9667
Δ-C22-Cer	S1P/T-Cer	-0.3835	0.9667
Δ-C22-Cer	S1P/C16	-0.3790	0.9667
Δ-S1P/C16-Cer	TOS [mcmol/l]	-0.3750	0.9667
Δ-C22-Cer	S1P/C24	-0.3402	0.9667
Δ-S1P/Tot-Cer	TOS [mcmol/l]	-0.3390	0.9667
Δ-C24-1-Cer	S1P/C14	-0.3358	0.9667
Δ-S1P/C18-Cer	C22-Cer	-0.3328	0.9667
Δ-Sph	S1P/C24-1	-0.3311	0.9667
Δ-C24-1-Cer	S1P	-0.3291	0.9667
Δ-S1P/C20-Cer	C22-Cer	-0.3257	0.9667
Δ-S1P/C14-Cer	S1P/C24	-0.3244	0.9667
Δ-C22-Cer	C20-Cer	0.3223	0.9667
Δ-S1P/C22-Cer	C22-Cer	-0.3221	0.9667
Δ-C20-Cer	TOS [mcmol/l]	0.3195	0.9667

Table S14. Multicollinearity diagnostics for ratio variables. Assessment of redundancy and shared information among sphingolipid ratio variables included in the ratio-only feature block. Analyses were performed on paired tumor–normal remodeling indices expressed as Δ values, where each variable represents the within-patient change in a sphingosine-1-phosphate (S1P)-to-ceramide ratio between tumor tissue and matched normal mucosa. "Max |ρ|" indicates the largest absolute pairwise correlation between a given ratio and any other ratio variable, whereas "Mean |ρ|" represents the mean absolute correlation with all remaining ratio variables and reflects average redundancy within the block. "R^2 on other ratios" denotes the proportion of variance in a given ratio explained by regression on all other ratio variables. The variance inflation factor (VIF) quantifies the degree to which collinearity inflates variance estimates in multivariable models, with larger values indicating greater redundancy and lower independent information content. The "Interpretive flag" provides a practical summary of the degree of multicollinearity. In this cohort, the highest VIF values were observed for Δ(S1P/Tot-Cer) and Δ(S1P/C16-Cer), indicating substantial overlap with the remaining ratio variables, whereas several other ratios showed only moderate or acceptable levels of multicollinearity. These findings demonstrate that redundancy within the ratio feature space was concentrated in selected variables rather than being uniform across the entire ratio block. Together with the minimal-panel analysis reported in Table S9, this pattern supports dimensional reduction to a representative ratio panel while reducing redundancy. Abbreviations: Δ, paired tumor-minus-normal change; ρ, correlation coefficient; R^2, coefficient of determination; VIF, variance inflation factor; S1P, sphingosine-1-phosphate; Cer, ceramide; Tot-Cer, total ceramides.

Variable	Max |ρ|	Mean |ρ|	R^2 on other ratios	VIF	Interpretive flag
Δ-S1P/Tot-Cer	0.9877	0.7330	0.9981	538.69	high
Δ-S1P/C16-Cer	0.9877	0.6760	0.9973	372.69	high
Δ-S1P/C24-1-Cer	0.7732	0.7192	0.9423	17.318	high
Δ-S1P/C24-Cer	0.7926	0.6859	0.8518	6.750	moderate
Δ-S1P/C22-Cer	0.7564	0.6371	0.7247	3.632	acceptable
Δ-S1P/C18-Cer	0.8084	0.6446	0.7048	3.388	acceptable
Δ-S1P/C20-Cer	0.7408	0.6040	0.6814	3.138	acceptable
Δ-S1P/C18-1-Cer	0.7551	0.6300	0.6317	2.715	acceptable

Table S15. **Genome-wide transcriptomic associations with anatomical position along the cecum-to-rectum axis in colorectal cancer.** This supplementary table provides an integrated summary of genome-wide transcriptomic associations with ordered colorectal anatomical position in 374 TCGA colorectal cancer samples mapped across an eight-level cecum-to-rectum axis. Across 19,885 analyzed genes, 3,376 genes showed significant associations with anatomical position, corresponding to 16.98% of the analyzed transcriptome. The table summarizes cohort geometry, PCA-based transcriptomic organization, position-associated gene rankings, directional asymmetry, curated biological modules, and ORA- and GSEA-derived functional programs. These results describe gene-expression associations with ordered anatomical position and should not be interpreted as evidence of a uniform continuous gradient across consecutive colorectal segments or as spatial transcriptomics at cellular or tissue-microdomain resolution.

S15A. Anatomical distribution of the TCGA CRC cohort

Spatial order represents the ordered anatomical position from cecum (1) to rectum (8).

Anatomical location	Number of cases	Spatial order	Percent of cohort
Cecum	78	1	20.86
Ascending Colon	61	2	16.31
Hepatic Flexure	17	3	4.55
Transverse Colon	23	4	6.15
Splenic Flexure	4	5	1.07
Descending Colon	15	6	4.01
Sigmoid Colon	83	7	22.19
Rectosigmoid Junction	1	7	0.27
Rectum	92	8	24.6

S15B. Global statistics of transcriptomic associations with anatomical position

Position-associated genes were identified using concordant statistical filtering and QC-adjusted modeling.

Metric	Value	Percent of transcriptome
Total genes analyzed	19,885	100
Position-associated genes	3376	16.98
Distal program genes	2584	12.99
Proximal program genes	792	3.98
Distal / proximal ratio	3.263	
PC2 Spearman rho with anatomical axis	0.405	
PC2 Spearman p value	3.54×10^{-16}	

S15C. PCA-derived transcriptomic association with anatomical position

PC2 showed a significant association with ordered anatomical position along the cecum-to-rectum axis.

Principal component	Spearman rho with spatial order	P value
PC2	0.405	3.54×10^{-16}

S15D. Exported position-associated transcriptomic inventory

Metric	Value
Total genes tested	19,885
Position-associated genes	3376
Distal increasing robust genes	2584
Proximal increasing robust genes	792
Tier 0 elite genes	9
Tier 0 + Tier 1 core genes	54
Top distal program genes exported	100
Top proximal program genes exported	100

S15E. Position-associated gene hierarchy by tier and direction

Tier classes stratify genes according to the robustness and magnitude of their association with anatomical position; Tier 0 represents genes with the strongest and most robust position-associated expression patterns.

Association tier	Direction	Number of genes
Tier 0 Elite	Distal increasing cecum-to-rectum	7
Tier 0 Elite	Proximal increasing rectum-to-cecum	2

Association tier	Direction	Number of genes
Tier 1 Core	Distal increasing cecum-to-rectum	41
Tier 1 Core	Proximal increasing rectum-to-cecum	4
Tier 2 Strong	Distal increasing cecum-to-rectum	527
Tier 2 Strong	Proximal increasing rectum-to-cecum	56
Tier 3 Robust FDR05	Distal increasing cecum-to-rectum	2009
Tier 3 Robust FDR05	Proximal increasing rectum-to-cecum	730

S15F. Curated biological module distribution among position-associated genes

Curated module	Distal-increasing genes	Proximal-increasing genes	Total	Distal/proximal ratio
Other or unclassified	2168	661	2829	3.28
Unresolved ENSG identifier	170	45	215	3.78
Zinc finger transcriptional regulation	130	17	147	7.65
Metabolic transport detoxification	65	20	85	3.25
Developmental patterning	12	26	38	0.46
Mucosal barrier epithelial identity	17	7	24	2.43
Immune inflammatory	4	10	14	0.4
Proliferation cell cycle	11	3	14	3.67
Invasion stromal EMT ECM	7	4	11	1.75

S15G. Top 50 genes with distal-increasing expression along the anatomical axis
Genes are ranked by priority score among genes showing distal-increasing expression along the cecum-to-rectum anatomical axis.

Gene	Direction	Tier	Curated module	Spearman rho	QC-adjusted beta	QC-adjusted FDR	Adjusted R2	Priority score
PRAC1	Distal increasing cecum-to-rectum	Tier 0 Elite	Other or unclassified	0.564	5.025	1.72×10^{-32}	0.365	90.06
LY6G6D	Distal increasing cecum-to-rectum	Tier 1 Core	Immune inflammatory	0.393	3.124	3.60×10^{-14}	0.199	16.49
ENSG00000242407	Distal increasing cecum-to-rectum	Tier 0 Elite	Unresolved ENSG identifier	0.511	1.708	5.77×10^{-18}	0.255	15.05
LY6G6F-LY6G6D	Distal increasing cecum-to-rectum	Tier 0 Elite	Immune inflammatory	0.406	2.167	1.36×10^{-14}	0.194	12.19
MAP7D2	Distal increasing cecum-to-rectum	Tier 0 Elite	Other or unclassified	0.43	1.505	1.65×10^{-12}	0.203	7.63
SYNE4	Distal increasing cecum-to-rectum	Tier 1 Core	Other or unclassified	0.385	1.539	8.53×10^{-13}	0.179	7.16
MUC12	Distal increasing cecum-to-rectum	Tier 1 Core	Mucosal barrier epithelial identity	0.381	1.818	1.42×10^{-10}	0.173	6.81
R3HDML-AS1	Distal increasing cecum-to-rectum	Tier 1 Core	Other or unclassified	0.398	1.417	1.29×10^{-12}	0.198	6.71
GNG4	Distal increasing cecum-to-rectum	Tier 2 Strong	Other or unclassified	0.339	1.863	5.04×10^{-10}	0.155	5.86
QPRT	Distal increasing cecum-to-rectum	Tier 2 Strong	Other or unclassified	0.348	1.558	4.24×10^{-10}	0.162	5.08
HOXB13	Distal increasing cecum-to-rectum	Tier 2 Strong	Developmental patterning	0.348	1.345	4.18×10^{-11}	0.172	4.86

Gene	Direction	Tier	Curated module	Spearman rho	QC-adjusted beta	QC-adjusted FDR	Adjusted R2	Priority score
ENSG00000250829	Distal increasing cecum-to-rectum	Tier 2 Strong	Unresolved ENSG identifier	0.334	1.567	2.65×10^{-9}	0.117	4.49
TNNC2	Distal increasing cecum-to-rectum	Tier 2 Strong	Other or unclassified	0.343	1.468	1.22×10^{-9}	0.134	4.49
ZNF880	Distal increasing cecum-to-rectum	Tier 1 Core	Zinc finger transcriptional regulation	0.377	0.938	6.90×10^{-12}	0.27	3.94
POFUT1	Distal increasing cecum-to-rectum	Tier 0 Elite	Other or unclassified	0.409	0.818	2.16×10^{-12}	0.323	3.9
ZNF813	Distal increasing cecum-to-rectum	Tier 0 Elite	Zinc finger transcriptional regulation	0.421	0.68	2.59×10^{-14}	0.228	3.9
KRT23	Distal increasing cecum-to-rectum	Tier 2 Strong	Mucosal barrier epithelial identity	0.307	1.84	3.07×10^{-7}	0.095	3.68
SPAG16	Distal increasing cecum-to-rectum	Tier 1 Core	Other or unclassified	0.352	1.088	7.06×10^{-10}	0.189	3.5
VAV3	Distal increasing cecum-to-rectum	Tier 2 Strong	Other or unclassified	0.345	1.263	1.53×10^{-8}	0.195	3.4
DIO3OS	Distal increasing cecum-to-rectum	Tier 2 Strong	Other or unclassified	0.322	1.239	5.08×10^{-9}	0.144	3.31
PFDN4	Distal increasing cecum-to-rectum	Tier 1 Core	Other or unclassified	0.386	0.754	2.56×10^{-11}	0.185	3.08
EREG	Distal increasing cecum-to-rectum	Tier 2 Strong	Other or unclassified	0.296	1.467	8.41×10^{-8}	0.115	3.08
GGT7	Distal increasing cecum-to-rectum	Tier 2 Strong	Metabolic transport detoxification	0.342	0.932	3.24×10^{-10}	0.282	3.02
TCEA2	Distal increasing cecum-to-rectum	Tier 1 Core	Other or unclassified	0.365	0.898	7.56×10^{-10}	0.286	2.99
ZNF528	Distal increasing cecum-to-rectum	Tier 1 Core	Zinc finger transcriptional regulation	0.386	0.811	4.58×10^{-10}	0.232	2.92
TP53RK	Distal increasing cecum-to-rectum	Tier 1 Core	Other or unclassified	0.386	0.677	7.91×10^{-12}	0.212	2.9
AOAH	Distal increasing cecum-to-rectum	Tier 2 Strong	Other or unclassified	0.324	1.13	1.44×10^{-8}	0.198	2.87
RUBCNL	Distal increasing cecum-to-rectum	Tier 2 Strong	Other or unclassified	0.316	1.263	1.05×10^{-7}	0.154	2.78
SYN3	Distal increasing	Tier 1 Core	Other or unclassified	0.358	1.028	2.84×10^{-8}	0.135	2.78

Gene	Direction	Tier	Curated module	Spearman rho	QC-adjusted beta	QC-adjusted FDR	Adjusted R2	Priority score
	cecum-to-rectum							
LOC101928881	Distal increasing cecum-to-rectum	Tier 2 Strong	Other or unclassified	0.341	0.893	3.17×10^{-9}	0.114	2.59
ASCL2	Distal increasing cecum-to-rectum	Tier 2 Strong	Other or unclassified	0.304	1.192	9.88×10^{-8}	0.097	2.54
GGH	Distal increasing cecum-to-rectum	Tier 2 Strong	Other or unclassified	0.333	0.979	1.64×10^{-8}	0.104	2.54
TSPAN6	Distal increasing cecum-to-rectum	Tier 1 Core	Other or unclassified	0.351	0.84	2.73×10^{-9}	0.143	2.53
LOC102723878	Distal increasing cecum-to-rectum	Tier 2 Strong	Other or unclassified	0.27	1.38	1.97×10^{-7}	0.107	2.5
DDX27	Distal increasing cecum-to-rectum	Tier 1 Core	Other or unclassified	0.378	0.673	1.75×10^{-10}	0.335	2.48
PLAGL2	Distal increasing cecum-to-rectum	Tier 1 Core	Other or unclassified	0.366	0.756	1.22×10^{-9}	0.301	2.46
SLC13A3	Distal increasing cecum-to-rectum	Tier 2 Strong	Metabolic transport detoxification	0.328	1.115	2.18×10^{-7}	0.1	2.44
MOCS3	Distal increasing cecum-to-rectum	Tier 1 Core	Other or unclassified	0.393	0.569	1.73×10^{-11}	0.268	2.41
GPR143	Distal increasing cecum-to-rectum	Tier 2 Strong	Other or unclassified	0.314	1.057	6.35×10^{-8}	0.133	2.39
INSL5	Distal increasing cecum-to-rectum	Tier 1 Core	Other or unclassified	0.366	0.819	1.16×10^{-8}	0.142	2.38
CHN2	Distal increasing cecum-to-rectum	Tier 1 Core	Other or unclassified	0.355	0.882	3.32×10^{-8}	0.161	2.34
ZNF347	Distal increasing cecum-to-rectum	Tier 0 Elite	Zinc finger transcriptional regulation	0.405	0.52	9.15×10^{-12}	0.3	2.32
PIGU	Distal increasing cecum-to-rectum	Tier 1 Core	Other or unclassified	0.366	0.613	4.80×10^{-11}	0.227	2.31
APCDD1	Distal increasing cecum-to-rectum	Tier 2 Strong	Other or unclassified	0.289	1.323	1.78×10^{-6}	0.105	2.2
PCSK1N	Distal increasing cecum-to-rectum	Tier 1 Core	Other or unclassified	0.365	0.989	1.09×10^{-6}	0.14	2.15
PCP4	Distal increasing cecum-to-rectum	Tier 2 Strong	Other or unclassified	0.301	1.282	2.70×10^{-6}	0.111	2.14

Gene	Direction	Tier	Curated module	Spearman rho	QC-adjusted beta	QC-adjusted FDR	Adjusted R2	Priority score
FITM2	Distal increasing cecum-to-rectum	Tier 1 Core	Other or unclassified	0.359	0.666	1.12×10^{-9}	0.245	2.14
MTERF1	Distal increasing cecum-to-rectum	Tier 2 Strong	Other or unclassified	0.338	0.734	2.37×10^{-9}	0.187	2.14
BEX2	Distal increasing cecum-to-rectum	Tier 2 Strong	Other or unclassified	0.313	1.123	9.84×10^{-7}	0.127	2.11
ENSG00000262370	Distal increasing cecum-to-rectum	Tier 2 Strong	Unresolved ENSG identifier	0.342	0.853	6.35×10^{-8}	0.149	2.1

S15H. Top 50 genes with proximal-increasing expression along the anatomical axis
Genes are ranked by priority score among genes showing proximal-increasing expression along the rectum-to-cecum anatomical axis.

Gene	Direction	Tier	Curated module	Spearman rho	QC-adjusted beta	5.60×10^{-13}	Adjusted R2	Priority score
ARHGAP4	Proximal increasing rectum-to-cecum	Tier 1 Core	Other or unclassified	-0.374	-1.415	2.38×10^{-13}	0.213	6.48
HOXC6	Proximal increasing rectum-to-cecum	Tier 0 Elite	Developmental patterning	-0.479	-0.963	4.03×10^{-11}	0.185	5.82
FOXD1	Proximal increasing rectum-to-cecum	Tier 1 Core	Developmental patterning	-0.359	-1.038	1.75×10^{-11}	0.173	3.87
EMX1	Proximal increasing rectum-to-cecum	Tier 0 Elite	Developmental patterning	-0.407	-0.849	2.70×10^{-9}	0.153	3.72
FOXD1-AS1	Proximal increasing rectum-to-cecum	Tier 1 Core	Developmental patterning	-0.369	-1.03	4.07×10^{-9}	0.145	3.26
DUSP4	Proximal increasing rectum-to-cecum	Tier 2 Strong	Other or unclassified	-0.282	-1.246	2.73×10^{-9}	0.158	2.94
FEZF1-AS1	Proximal increasing rectum-to-cecum	Tier 2 Strong	Other or unclassified	-0.329	-1.042	4.24×10^{-10}	0.131	2.93
TFAP2A	Proximal increasing rectum-to-cecum	Tier 2 Strong	Developmental patterning	-0.311	-0.963	8.75×10^{-12}	0.173	2.81
PAX9	Proximal increasing rectum-to-cecum	Tier 1 Core	Developmental patterning	-0.399	-0.582	3.88×10^{-8}	0.172	2.57
TRIM7	Proximal increasing rectum-to-cecum	Tier 2 Strong	Other or unclassified	-0.269	-1.179	8.99×10^{-8}	0.141	2.35
FOXA1	Proximal increasing rectum-to-cecum	Tier 2 Strong	Developmental patterning	-0.307	-1.017	8.68×10^{-8}	0.121	2.2

Gene	Direction	Tier	Curated module	Spearman rho	QC-adjusted beta	5.60×10^{-13}	Adjusted R2	Priority score
BST2	Proximal increasing rectum-to-cecum	Tier 3 Robust FDR05	Other or unclassified	-0.248	-1.216	7.56×10^{-10}	0.194	2.12
RAB27B	Proximal increasing rectum-to-cecum	Tier 2 Strong	Other or unclassified	-0.286	-0.811	1.64×10^{-8}	0.163	2.12
GAD1	Proximal increasing rectum-to-cecum	Tier 2 Strong	Other or unclassified	-0.307	-0.841	5.57×10^{-6}	0.122	2.01
CTSE	Proximal increasing rectum-to-cecum	Tier 3 Robust FDR05	Other or unclassified	-0.227	-1.592	8.89×10^{-5}	0.098	1.9
REG4	Proximal increasing rectum-to-cecum	Tier 3 Robust FDR05	Other or unclassified	-0.217	-2.143	1.14×10^{-6}	0.071	1.89
HOXB6	Proximal increasing rectum-to-cecum	Tier 2 Strong	Developmental patterning	-0.308	-0.978	3.40×10^{-8}	0.087	1.79
ADGRG6	Proximal increasing rectum-to-cecum	Tier 2 Strong	Other or unclassified	-0.293	-0.813	7.89×10^{-9}	0.157	1.78
DAPK1	Proximal increasing rectum-to-cecum	Tier 3 Robust FDR05	Other or unclassified	-0.23	-0.884	2.68×10^{-7}	0.226	1.64
MYRF	Proximal increasing rectum-to-cecum	Tier 2 Strong	Other or unclassified	-0.256	-0.962	6.42×10^{-8}	0.161	1.62
DNAAF3	Proximal increasing rectum-to-cecum	Tier 2 Strong	Other or unclassified	-0.336	-0.627	9.04×10^{-7}	0.152	1.52
FEZF1	Proximal increasing rectum-to-cecum	Tier 2 Strong	Other or unclassified	-0.293	-0.788	1.10×10^{-5}	0.101	1.39
TNNT1	Proximal increasing rectum-to-cecum	Tier 2 Strong	Other or unclassified	-0.271	-1.036	5.13×10^{-7}	0.129	1.39
HAGLR	Proximal increasing rectum-to-cecum	Tier 2 Strong	Other or unclassified	-0.256	-0.859	1.30×10^{-7}	0.125	1.38
EXOC3L4	Proximal increasing rectum-to-cecum	Tier 2 Strong	Other or unclassified	-0.291	-0.657	2.37×10^{-9}	0.128	1.32
HOXB2	Proximal increasing rectum-to-cecum	Tier 3 Robust FDR05	Developmental patterning	-0.199	-0.765	6.12×10^{-6}	0.24	1.31
GABRP	Proximal increasing rectum-to-cecum	Tier 2 Strong	Other or unclassified	-0.25	-0.958	3.15×10^{-5}	0.094	1.25
LYZ	Proximal increasing rectum-to-cecum	Tier 3 Robust FDR05	Other or unclassified	-0.208	-1.328	5.44×10^{-7}	0.095	1.24
NXF3	Proximal increasing	Tier 2 Strong	Other or unclassified	-0.264	-0.749	7.89×10^{-9}	0.094	1.24

Gene	Direction	Tier	Curated module	Spearman rho	QC-adjusted beta	5.60 × 10⁻¹³	Adjusted R2	Priority score
	rectum-to-cecum							
FAM72C	Proximal increasing rectum-to-cecum	Tier 2 Strong	Other or unclassified	-0.29	-0.522	1.18×10^{-6}	0.149	1.23
SLC28A3	Proximal increasing rectum-to-cecum	Tier 2 Strong	Metabolic transport detoxification	-0.268	-0.764	6.23×10^{-7}	0.084	1.21
RPL32P29	Proximal increasing rectum-to-cecum	Tier 2 Strong	Other or unclassified	-0.31	-0.628	3.44×10^{-6}	0.173	1.21
TRNP1	Proximal increasing rectum-to-cecum	Tier 3 Robust FDR05	Other or unclassified	-0.231	-0.929	5.04×10^{-10}	0.119	1.17
HOXD3	Proximal increasing rectum-to-cecum	Tier 2 Strong	Developmental patterning	-0.344	-0.364	5.12×10^{-6}	0.128	1.16
AGR2	Proximal increasing rectum-to-cecum	Tier 3 Robust FDR05	Other or unclassified	-0.237	-0.9	6.27×10^{-8}	0.15	1.13
KCTD1	Proximal increasing rectum-to-cecum	Tier 3 Robust FDR05	Other or unclassified	-0.247	-0.625	6.91×10^{-7}	0.201	1.11
OASL	Proximal increasing rectum-to-cecum	Tier 3 Robust FDR05	Other or unclassified	-0.243	-0.741	6.77×10^{-8}	0.149	1.11
TRIB2	Proximal increasing rectum-to-cecum	Tier 3 Robust FDR05	Other or unclassified	-0.209	-0.731	9.58×10^{-8}	0.228	1.1
CIITA	Proximal increasing rectum-to-cecum	Tier 3 Robust FDR05	Other or unclassified	-0.184	-0.829	1.81×10^{-5}	0.185	1.07
DEGS2	Proximal increasing rectum-to-cecum	Tier 2 Strong	Other or unclassified	-0.285	-0.769	5.86×10^{-7}	0.125	1.04
ENSG00000254802	Proximal increasing rectum-to-cecum	Tier 2 Strong	Unresolved ENSG identifier	-0.317	-0.525	5.40×10^{-8}	0.087	1.04
MIR5689HG	Proximal increasing rectum-to-cecum	Tier 2 Strong	Other or unclassified	-0.292	-0.483	1.05×10^{-5}	0.134	1.02
PLAAT4	Proximal increasing rectum-to-cecum	Tier 3 Robust FDR05	Other or unclassified	-0.221	-0.913	4.97×10^{-8}	0.151	1.01
SAMD9L	Proximal increasing rectum-to-cecum	Tier 3 Robust FDR05	Other or unclassified	-0.199	-0.69	5.17×10^{-6}	0.265	1
AMH	Proximal increasing rectum-to-cecum	Tier 2 Strong	Other or unclassified	-0.257	-0.721	6.72×10^{-8}	0.072	0.98
OTX1	Proximal increasing rectum-to-cecum	Tier 2 Strong	Other or unclassified	-0.267	-0.51	6.88×10^{-7}	0.156	0.98

Gene	Direction	Tier	Curated module	Spearman rho	QC-adjusted beta	5.60 × 10^{-13}	Adjusted R2	Priority score
DAPP1	Proximal increasing rectum-to-cecum	Tier 3 Robust FDR05	Other or unclassified	-0.232	-0.682	1.45 × 10^{-7}	0.149	0.98
PNPLA3	Proximal increasing rectum-to-cecum	Tier 2 Strong	Other or unclassified	-0.302	-0.469	3.72 × 10^{-6}	0.109	0.97
CCL5	Proximal increasing rectum-to-cecum	Tier 3 Robust FDR05	Immune inflammatory	-0.199	-0.876	6.58 × 10^{-6}	0.198	0.95
IQGAP2	Proximal increasing rectum-to-cecum	Tier 2 Strong	Other or unclassified	-0.26	-0.694	5.60 × 10^{-13}	0.083	0.94

S15I. Functional programs associated with anatomical position along the cecum-to-rectum axis

Anatomical enrichment	Functional program	Biological interpretation
Proximal CRC / cecum-enriched	Interferon gamma and alpha response	Immune-associated proximal tumor profile
Proximal CRC / cecum-enriched	Antigen processing and presentation	Enhanced antigen-presentation machinery
Proximal CRC / cecum-enriched	T-cell and NK-cell activation	Adaptive and innate immune engagement
Proximal CRC / cecum-enriched	Developmental/anterior-posterior patterning	Retention of axial developmental memory
Proximal CRC / cecum-enriched	Death receptor/apoptotic signaling	Increased death-receptor and apoptosis-associated signaling
Distal CRC / rectum-enriched	MYC targets	Biosynthetic and growth-associated distal tumor profile
Distal CRC / rectum-enriched	Ribosome biogenesis	Enhanced translational machinery
Distal CRC / rectum-enriched	rRNA processing	Increased ribosomal RNA maturation
Distal CRC / rectum-enriched	RNA polymerase / transcription	Increased transcriptional output
Distal CRC / rectum-enriched	mRNA splicing and RNA transport	Post-transcriptional RNA-processing amplification

S15J. ORA theme-level summary of significant functional enrichment

Direction	Functional theme	Number of ORA terms, FDR < 0.05
Distal enriched cecum-to-rectum	RNA processing transport transcription	3
Distal enriched cecum-to-rectum	RNA processing transport transcription; Metabolism transport	2
Distal enriched cecum-to-rectum	Immune T cell antigen interferon	1
Distal enriched cecum-to-rectum	MYC cell cycle growth	1
Proximal enriched rectum-to-cecum	Immune T cell antigen interferon	14
Proximal enriched rectum-to-cecum	Other	8
Proximal enriched rectum-to-cecum	Developmental patterning morphogenesis	4
Proximal enriched rectum-to-cecum	Apoptosis death receptor	2
Proximal enriched rectum-to-cecum	Metabolism transport	2

S15K. GSEA theme-level summary of significant functional enrichment

Direction	Functional theme	Number of GSEA terms, FDR < 0.05
Enriched distal cecum-to-rectum	Other	45
Enriched distal cecum-to-rectum	Ribosome rRNA translation	22
Enriched distal cecum-to-rectum	RNA processing transport transcription	15
Enriched distal cecum-to-rectum	Metabolism transport	5

Direction	Functional theme	Number of GSEA terms, FDR < 0.05
Enriched distal cecum-to-rectum	Ribosome rRNA translation; RNA processing transport transcription	4
Enriched distal cecum-to-rectum	MYC cell cycle growth	2
Enriched distal cecum-to-rectum	Immune T cell antigen interferon; RNA processing transport transcription	1
Enriched distal cecum-to-rectum	Immune T cell antigen interferon; Ribosome rRNA translation;RNA processing transport transcription	1
Enriched distal cecum-to-rectum	RNA processing transport transcription; Metabolism transport	1
Enriched proximal rectum-to-cecum	Other	134
Enriched proximal rectum-to-cecum	Immune T cell antigen interferon	113
Enriched proximal rectum-to-cecum	Metabolism transport	9
Enriched proximal rectum-to-cecum	Immune T cell antigen interferon; ECM invasion stromal	7
Enriched proximal rectum-to-cecum	Immune T cell antigen interferon; MYC cell cycle growth	6
Enriched proximal rectum-to-cecum	Apoptosis death receptor	3
Enriched proximal rectum-to-cecum	Developmental patterning morphogenesis	3
Enriched proximal rectum-to-cecum	MYC cell cycle growth	2
Enriched proximal rectum-to-cecum	ECM invasion stromal	1
Enriched proximal rectum-to-cecum	RNA processing transport transcription	1

S15L. GSEA normalized enrichment score summary by functional theme

Direction	Functional theme	Terms	Median NES	Maximum absolute NES	Minimum FDR
Enriched distal cecum-to-rectum	MYC cell cycle growth	2	2.135	2.238	0
Enriched distal cecum-to-rectum	Other	45	1.879	2.29	0
Enriched distal cecum-to-rectum	Ribosome rRNA translation	22	2.012	2.421	0
Enriched distal cecum-to-rectum	Ribosome rRNA translation; RNA processing transport transcription	4	2.341	2.36	0
Enriched distal cecum-to-rectum	RNA processing transport transcription	15	1.969	2.172	0
Enriched distal cecum-to-rectum	Metabolism transport	5	1.88	2.096	0.01
Enriched distal cecum-to-rectum	RNA processing transport transcription; Metabolism transport	1	1.89	1.89	0.01
Enriched distal cecum-to-rectum	Immune T cell antigen interferon;Ribosome rRNA translation; RNA processing transport transcription	1	1.843	1.843	0.03
Enriched distal cecum-to-rectum	Immune T cell antigen interferon; RNA processing transport transcription	1	1.855	1.855	0.03
Enriched proximal rectum-to-cecum	Developmental patterning morphogenesis	3	-1.992	2.196	0
Enriched proximal rectum-to-cecum	Immune T cell antigen interferon	113	-2.075	3.204	0
Enriched proximal rectum-to-cecum	Immune T cell antigen interferon; MYC cell cycle growth	6	-2.055	2.316	0
Enriched proximal rectum-to-cecum	Other	134	-1.936	2.984	0
Enriched proximal rectum-to-cecum	ECM invasion stromal	1	-1.96	1.96	0
Enriched proximal rectum-to-cecum	Immune T cell antigen interferon; ECM invasion stromal	7	-1.888	2.037	0.01
Enriched proximal rectum-to-cecum	MYC cell cycle growth	2	-1.768	2.017	0.01
Enriched proximal rectum-to-cecum	RNA processing transport transcription	1	-1.756	1.756	0.01

Direction	Functional theme	Terms	Median NES	Maximum absolute NES	Minimum FDR
Enriched proximal rectum-to-cecum	Metabolism transport	9	-1.778	1.889	0.02
Enriched proximal rectum-to-cecum	Apoptosis death receptor	3	-1.574	1.898	0.04

S15M. Top GSEA terms associated with anatomical position

Direction	Functional theme	Library	Term	NES	FDR	Leading-edge genes
Enriched distal cecum-to-rectum	MYC cell cycle growth	MSigDB Hallmark 2020	Myc Targets V1	2.238	0	EIF2S2; PSMA7; NCBP2; CBX3; SSB; SSBP1; EIF3B; MRPL9; UBA2; CUL1; +81 more
Enriched distal cecum-to-rectum	MYC cell cycle growth	MSigDB Hallmark 2020	Myc Targets V2	2.032	0	CBX3; PRMT3; MPHOSPH10; LAS1L; NIP7; HSPE1; EXOSC5; DDX18; NDUFAF4; RRP9; +15 more
Enriched distal cecum-to-rectum	Other	GO Cellular Component 2023	Small-Subunit Processome	2.29	0	UTP14A; DCAF13; UTP6; PNO1; NOL10; NAT10; MPHOSPH10; XRCC5; UTP25; UTP4; +34 more
Enriched distal cecum-to-rectum	Ribosome rRNA translation	GO Biological Process 2023	Ribosome Biogenesis	2.421	0	DDX27; PIN4; RBIS; DKC1; GTF3A; DCAF13; UTP6; PNO1; NAT10; MPHOSPH10; +71 more
Enriched distal cecum-to-rectum	Ribosome rRNA translation	GO Biological Process 2023	Ribonucleoprotein Complex Biogenesis	2.359	0	RBIS; GTF3A; UTP6; PNO1; NAT10; MPHOSPH10; UTP4; CUL4B; NVL; RPF2; +61 more
Enriched distal cecum-to-rectum	Ribosome rRNA translation	Reactome 2022	rRNA Modification In Nucleus And Cytosol	2.309	0	UTP14A; DKC1; DCAF13; UTP6; PNO1; NAT10; MPHOSPH10; UTP25; UTP4; NOL11; +25 more
Enriched distal cecum-to-rectum	Ribosome rRNA translation;RNA processing transport transcription	GO Biological Process 2023	rRNA Processing	2.343	0	DDX27; EIF6; PIN4; DKC1; DCAF13; NEDD4; NAT10; MPHOSPH10; LAS1L; UTP4; +53 more
Enriched distal cecum-to-rectum	Ribosome rRNA translation;RNA processing transport transcription	Reactome 2022	rRNA Processing In Nucleus And Cytosol	2.36	0	RPS21; UTP14A; DKC1; DCAF13; UTP6; RPL21; PNO1; DNAJC5; RPP40; NAT10; +96 more
Enriched distal cecum-to-rectum	Ribosome rRNA translation;RNA processing transport transcription	Reactome 2022	Major Pathway Of rRNA Processing In Nucleolus And Cytosol	2.338	0	RPS21; UTP14A; DCAF13; UTP6; RPL21; PNO1; DNAJC5; RPP40; MPHOSPH10; LAS1L; +91 more
Enriched distal cecum-to-rectum	Ribosome rRNA translation;RNA processing transport transcription	Reactome 2022	rRNA Processing	2.304	0	RPS21; UTP14A; DKC1; DCAF13; UTP6; RPL21; PNO1; DNAJC5; RPP40; NAT10; +101 more
Enriched proximal rectum-to-cecum	Developmental patterning morphogenesis	GO Biological Process 2023	Embryonic Skeletal System Morphogenesis	-2.196	0	TFAP2A; HOXD3; HOXB4; HOXD4; HOXC4; OSR2; FGFR2; HOXB3
Enriched proximal rectum-to-cecum	Immune T cell antigen interferon	GO Biological Process 2023	Regulation Of Immune Response	-2.539	0	PDCD1; LAG3; SLA2; CXCL13; IL15; HLA-DRB1; IRF7; SH2D1A;

Direction	Functional theme	Library	Term	NES	FDR	Leading-edge genes
						BCL6; HLA-DRA; +25 more
Enriched proximal rectum-to-cecum	Immune T cell antigen interferon	GO Biological Process 2023	T Cell Activation	-2.485	0	NLRC3; CD8A; SLA2; RASGRP1; TRGC2; CD7; ITK; PIK3CD; LCK; CD247; +35 more
Enriched proximal rectum-to-cecum	Immune T cell antigen interferon	GO Biological Process 2023	Antigen Receptor-Mediated Signaling Pathway	-2.443	0	PDE4D; BTNL9; CD8A; SLA2; TXK; TRGC2; ITK; PIK3CD; PRKCB; LCK; +38 more
Enriched proximal rectum-to-cecum	Immune T cell antigen interferon	GO Biological Process 2023	T Cell Receptor Signaling Pathway	-2.415	0	PDE4D; BTNL9; CD8A; TXK; TRGC2; ITK; PIK3CD; LCK; CD247; ZAP70; +33 more
Enriched proximal rectum-to-cecum	Immune T cell antigen interferon	GO Biological Process 2023	Immunoglobulin Mediated Immune Response	-2.396	0	HLA-DMA; IL2RB; HLA-DRB1; CD74; IL21R; HLA-DPA1; HLA-DRA; HLA-DMB; HLA-DOA; HLA-DRB5; +9 more
Enriched proximal rectum-to-cecum	Immune T cell antigen interferon;MYC cell cycle growth	GO Biological Process 2023	Regulation Of T Cell Proliferation	-2.316	0	CCL5; JAK2; TNFRSF13C; TNFSF9; IL15; CD3E; HLA-DRB1; CD6; TNFSF8; HLA-DPA1; +24 more
Enriched proximal rectum-to-cecum	Other	GO Biological Process 2023	Regulation Of Interleukin-2 Production	-2.293	0	PDE4D; HAVCR2; GBP1; PTPRC; VSIG4; LILRB4; PLCG2; CD28; CR1; LAPTM5; +17 more
Enriched proximal rectum-to-cecum	Other	GO Biological Process 2023	Negative Regulation Of Interleukin-10 Production	-2.243	0	TRIB2; VSIR; LILRB1; LILRB4; TYROBP; CD274; DLL1; FOXP3; FCGR2B; PDCD1LG2; +1 more
Enriched proximal rectum-to-cecum	Other	GO Biological Process 2023	Regulation Of Tumor Necrosis Factor Production	-2.228	0	NR1H4; NLRC3; CHRNA7; AMH; JAK2; RASGRP1; OAS2; CX3CL1; TMEM106A; CD2; +46 more
Enriched proximal rectum-to-cecum	Other	GO Biological Process 2023	Negative Regulation Of Interleukin-2 Production	-2.222	0	HAVCR2; GBP1; PTPRC; VSIG4; LILRB4; CR1; LAPTM5; EZR; FOXP3; ZFP36; +2 more
Enriched proximal rectum-to-cecum	Other	Reactome 2022	Immunoregulatory Interactions Between A Lymphoid And A non-Lymphoid Cell	-2.984	0	KLRK1; ITGB7; KIR2DL4; CD8A; KLRC1; CLEC2D; ITGAL; CD96; CD247; CD3E; +54 more
Enriched proximal rectum-to-cecum	Immune T cell antigen interferon;MYC cell cycle growth	GO Biological Process 2023	Positive Regulation Of Lymphocyte Proliferation	-2.162	0	CCL5; AMH; JAK2; TNFRSF13C; IL15; CD3E; CD74; CD6; BCL2; HLA-DPA1; +21 more
Enriched distal cecum-to-rectum	Ribosome rRNA translation	KEGG 2021 Human	Ribosome biogenesis in eukaryotes	2.106	0	EIF6; CSNK2A2; UTP14A; DKC1; UTP6; RPP40; NAT10; MPHOSPH10; UTP4; NVL; +36 more
Enriched proximal rectum-to-cecum	ECM invasion stromal	KEGG 2021 Human	Cell adhesion molecules	-1.96	0	PDCD1; ITGB7; CD8A; TIGIT; HLA-DMA; ITGAL; HLA-DRB1; HLA-DPB1; CD6; CD2; +41 more

Direction	Functional theme	Library	Term	NES	FDR	Leading-edge genes
Enriched proximal rectum-to-cecum	Immune T cell antigen interferon;MYC cell cycle growth	GO Biological Process 2023	Negative Regulation Of Lymphocyte Proliferation	-2.119	0	HLA-DRB1; CTLA4; LILRB1; PLA2G2D; DLG5; LYN; CD300A; VSIG4; LILRB4; CR1; +14 more
Enriched distal cecum-to-rectum	Ribosome rRNA translation	Reactome 2022	Translation	2.162	0	EIF2S2; RPN2; MRPS31; MTIF3; DARS1; RPS21; EIF3B; RPL21; MRPL9; PARS2; +120 more
Enriched distal cecum-to-rectum	RNA processing transport transcription	GO Biological Process 2023	ncRNA Processing	2.172	0	DDX27; PIN4; SSB; DKC1; DCAF13; NVL; RPP30; MRM2; NOL8; EXOSC5; +38 more
Enriched distal cecum-to-rectum	Other	KEGG 2021 Human	Peroxisome	2.024	0	ACOT8; PXMP4; AMACR; PIPOX; ACSL6; NUDT7; PHYH; PRDX5; ACSL5; NUDT12; +21 more
Enriched distal cecum-to-rectum	RNA processing transport transcription	Reactome 2022	mRNA Splicing - Minor Pathway	2.12	0	PRPF6; GTF2F2; NCBP2; POLR2K; SNRPE; NCBP1; SRSF6; POLR2F; POLR2J; SF3B6; +9 more

S15N. Top ORA terms associated with anatomical position

Direction	Functional theme	Library	Term	Overlap	FDR	Odds ratio	Genes
Proximal enriched rectum-to-cecum	Immune T cell antigen interferon	MSigDB Hallmark 2020	Interferon Gamma Response	39/200	6.60×10^{-16}	6.53	RTP4; SAMD9L; CIITA; NLRC5; IFI30; DDX60; USP18; IFIT3; OASL; HLA-DMA; +29 more
Proximal enriched rectum-to-cecum	Immune T cell antigen interferon	MSigDB Hallmark 2020	Interferon Alpha Response	23/97	2.07×10^{-11}	8.23	RTP4; CD74; SAMD9L; IL15; TAP1; GMPR; IFI44; ISG15; IFI30; DDX60; +13 more
Distal enriched cecum-to-rectum	Immune T cell antigen interferon	KEGG 2021 Human	Herpes simplex virus 1 infection	102/498	1.39×10^{-5}	1.92	ZNF175; ZNF570; ZFP30; ZNF606; ZNF85; ZNF569; ZNF568; IL12A; MAP3K7; ZNF443; +92 more
Distal enriched cecum-to-rectum	RNA processing transport transcription	Reactome 2022	Gene Expression (Transcription)	244/1449	2.95×10^{-5}	1.53	TDRKH; ZNF175; TP53RK; CCNH; HDAC11; UXT; PSMD6; PSMD7; HENMT1; ZNF606; +234 more
Proximal enriched rectum-to-cecum	Immune T cell antigen interferon	KEGG 2021 Human	Antigen processing and presentation	14/78	2.98×10^{-4}	5.73	CD74; CIITA; TAP1; IFI30; KIR2DL4; TAPBP; HLA-DMA; CD8A; PSME1; PSME2; +4 more
Distal enriched cecum-to-rectum	RNA processing transport transcription	Reactome 2022	Generic Transcription Pathway	198/1190	5.76×10^{-4}	1.49	ZNF175; TP53RK; CCNH; HDAC11; UXT; PSMD6;

Direction	Functional theme	Library	Term	Overlap	FDR	Odds ratio	Genes
							PSMD7; ZNF606; MYC; MYB; +188 more
Distal enriched cecum-to-rectum	RNA processing transport transcription	Reactome 2022	RNA Polymerase II Transcription	215/1312	5.76×10^{-4}	1.47	ZNF175; TP53RK; CCNH; HDAC11; UXT; PSMD6; PSMD7; ZNF606; MYC; MYB; +205 more
Proximal enriched rectum-to-cecum	Immune T cell antigen interferon	GO Biological Process 2023	Defense Response To Virus	23/189	0	3.65	RTP4; BNIP3L; IFI6; NLRC5; CGAS; DDX60L; ISG15; DDX60; PHB2; LYST; +13 more
Distal enriched cecum-to-rectum	RNA processing transport transcription;Metabolism transport	Reactome 2022	Metabolism Of RNA	117/666	0.01	1.58	LTV1; RPL30; TP53RK; CCNH; RPP30; TSEN2; TSEN54; RRP9; PSMD6; PSMD7; +107 more
Proximal enriched rectum-to-cecum	Developmental patterning morphogenesis	GO Biological Process 2023	Embryonic Skeletal System Morphogenesis	8/30	0.01	9.46	TFAP2A; OSR2; HOXB4; HOXC4; HOXB3; HOXD4; HOXD3; FGFR2
Proximal enriched rectum-to-cecum	Immune T cell antigen interferon	GO Biological Process 2023	Defense Response To Symbiont	18/148	0.01	3.63	RTP4; BNIP3L; IFI6; NLRC5; CGAS; DDX60L; ISG15; DDX60; LYST; IFIT3; +8 more
Proximal enriched rectum-to-cecum	Other	MSigDB Hallmark 2020	Allograft Rejection	18/200	0.01	2.59	CD74; IL15; GZMA; GALNT1; TAP1; CXCL13; WARS1; TAPBP; HLA-DMA; CD8A; +8 more
Distal enriched cecum-to-rectum	RNA processing transport transcription;Metabolism transport	KEGG 2021 Human	RNA transport	41/186	0.01	2.08	CYFIP2; NXT1; FMR1; RPP30; NXT2; NUP160; NUP42; MAGOH; XPO5; SAP18; +31 more
Proximal enriched rectum-to-cecum	Apoptosis death receptor	GO Molecular Function 2023	Death Receptor Activity	5/11	0.01	21.62	FAS; TNFRSF10B; TNFRSF10A; TNFRSF14; TNFRSF11A
Proximal enriched rectum-to-cecum	Other	MSigDB Hallmark 2020	TNF-alpha Signaling via NF-kB	17/200	0.02	2.43	DUSP4; TGIF1; TNFAIP8; GCH1; PLK2; RNF19B; TAP1; F3; SERPINB8; NR4A2; +7 more
Distal enriched cecum-to-rectum	MYC cell cycle growth	MSigDB Hallmark 2020	Myc Targets V2	17/58	0.02	3.03	DDX18; CBX3; NIP7; PRMT3; TFB2M; HSPE1; WDR43; RRP9; GNL3; HSPD1; +7 more
Proximal enriched rectum-to-cecum	Metabolism transport	MSigDB Hallmark 2020	heme Metabolism	16/200	0.02	2.27	BNIP3L; ALDH1L1; SLC66A2; FECH; MOCOS; AQP3; ANK1;

Direction	Functional theme	Library	Term	Overlap	FDR	Odds ratio	Genes
							RAP1GAP; EPOR; PSMD9; +6 more
Proximal enriched rectum-to-cecum	Other	MSigDB Hallmark 2020	Inflammatory Response	16/200	0.02	2.27	RTP4; GCH1; IL15; CXCR6; SGMS2; F3; RASGRP1; CX3CL1; TAPBP; BST2; +6 more
Proximal enriched rectum-to-cecum	Other	MSigDB Hallmark 2020	p53 Pathway	16/200	0.02	2.27	HEXIM1; PLK2; HSPA4L; RPS27L; RNF19B; TAP1; IFI30; SERPINB5; DDB2; DDIT4; +6 more
Proximal enriched rectum-to-cecum	Developmental patterning morphogenesis	GO Biological Process 2023	Anterior/Posterior Pattern Specification	10/59	0.03	5.32	HOXB4; HOXB3; HOXC4; HOXD4; HOXD3; HOXB7; HOXB6; HOXB5; HES4; HOXC6
Proximal enriched rectum-to-cecum	Other	Reactome 2022	Chemokine Receptors Bind Chemokines	10/56	0.04	5.67	CXCL11; CCL5; CCL4; XCL2; CXCR5; ACKR4; CXCR4; CXCR6; CXCL13; CX3CL1
Proximal enriched rectum-to-cecum	Apoptosis death receptor	GO Biological Process 2023	Apoptotic Process	21/228	0.05	2.66	IFI27L1; EGLN3; DAPK1; GZMA; IFI6; JADE1; CXCR4; TNFRSF10B; CIB1; TNFRSF10A; +11 more
Proximal enriched rectum-to-cecum	Metabolism transport	MSigDB Hallmark 2020	Glycolysis	15/200	0.05	2.11	EGLN3; GOT1; PGAM1; SRD5A3; CXCR4; ENO1; ENO2; ISG20; FUT8; PKM; +5 more
Proximal enriched rectum-to-cecum	Developmental patterning morphogenesis	GO Biological Process 2023	Skeletal System Morphogenesis	8/43	0.05	5.94	TFAP2A; OSR2; HOXB4; HOXC4; HOXB3; HOXD4; HOXD3; FGFR2
Proximal enriched rectum-to-cecum	Developmental patterning morphogenesis	GO Biological Process 2023	Embryonic Organ Morphogenesis	8/44	0.05	5.78	MESP1; OSR2; HOXB4; HOXC4; HOXB3; HOXD4; HOXD3; FGFR2

Table S16. Associations of sphingolipid-pathway gene expression with anatomical position along the cecum-to-rectum axis in the TCGA colorectal cancer cohort. Expression of genes involved in de novo sphingolipid synthesis, ceramide metabolism, sphingosine-1-phosphate (S1P) turnover, and S1P receptor signaling was correlated with ordered anatomical position along an eight-level cecum-to-rectum axis using Spearman correlation analysis. Multiple-testing correction was performed using the Benjamini–Hochberg false discovery rate (FDR) procedure. Positive Spearman ρ values indicate increasing expression toward distal anatomical positions, whereas negative values indicate increasing expression toward proximal anatomical positions.

Gene	Functional module	Spearman ρ	P-value	FDR q-value	Direction of association	FDR significance
SPTLC1	De novo synthesis	0.137	7.76×10^{-3}	0.0308	Distal-increasing	Yes
SPTLC2	De novo synthesis	−0.102	0.0484	0.0865	Proximal-increasing	No

Gene	Functional module	Spearman ρ	P-value	FDR q-value	Direction of association	FDR significance
SPTLC3	De novo synthesis	−0.190	2.22×10^{-4}	0.00170	Proximal-increasing	Yes
KDSR	De novo synthesis	−0.104	0.0448	0.0865	Proximal-increasing	No
DEGS1	Ceramide maturation	−0.001	0.981	0.981	No clear directional association	No
DEGS2	Ceramide maturation	−0.285	1.91×10^{-8}	4.40×10^{-7}	Proximal-increasing	Yes
CERS1	Ceramide synthesis	0.105	0.0424	0.0865	Distal-increasing	No
CERS2	Ceramide synthesis	0.172	8.10×10^{-4}	0.00466	Distal-increasing	Yes
CERS3	Ceramide synthesis	—	—	—	Not detected in expression matrix	Not tested
CERS4	Ceramide synthesis	0.208	5.22×10^{-5}	6.01×10^{-4}	Distal-increasing	Yes
CERS5	Ceramide synthesis	0.101	0.0519	0.0865	Distal-increasing	No
CERS6	Ceramide synthesis	0.094	0.0704	0.101	Distal-increasing	No
ASAH1	Ceramide degradation	−0.043	0.410	0.471	Proximal-increasing	No
ASAH2	Ceramide degradation	−0.054	0.294	0.356	Proximal-increasing	No
SPHK1	S1P synthesis	−0.079	0.128	0.173	Proximal-increasing	No
SPHK2	S1P synthesis	−0.024	0.638	0.699	No clear directional association	No
SGPP1	S1P turnover	−0.016	0.763	0.797	No clear directional association	No
SGPP2	S1P turnover	−0.137	8.04×10^{-3}	0.0308	Proximal-increasing	Yes
SGPL1	S1P degradation	−0.068	0.190	0.243	Proximal-increasing	No
S1PR1	S1P signaling	0.111	0.0323	0.0827	Distal-increasing	No
S1PR2	S1P signaling	−0.112	0.0308	0.0827	Proximal-increasing	No
S1PR3	S1P signaling	0.118	0.0219	0.0721	Distal-increasing	No
S1PR4	S1P signaling	−0.098	0.0592	0.0907	Proximal-increasing	No
S1PR5	S1P signaling	—	—	—	Not detected in expression matrix	Not tested
ENPP2	S1P-related lipid signaling	0.100	0.0526	0.0865	Distal-increasing	No

Abbreviations

Abbreviation list

Abbreviation	Definition
CRC	colorectal cancer
TCGA	The Cancer Genome Atlas
PCA	principal component analysis
PC2	second principal component
FDR	false discovery rate
ORA	over-representation analysis
GSEA	gene set enrichment analysis
NES	normalized enrichment score
QC	quality control

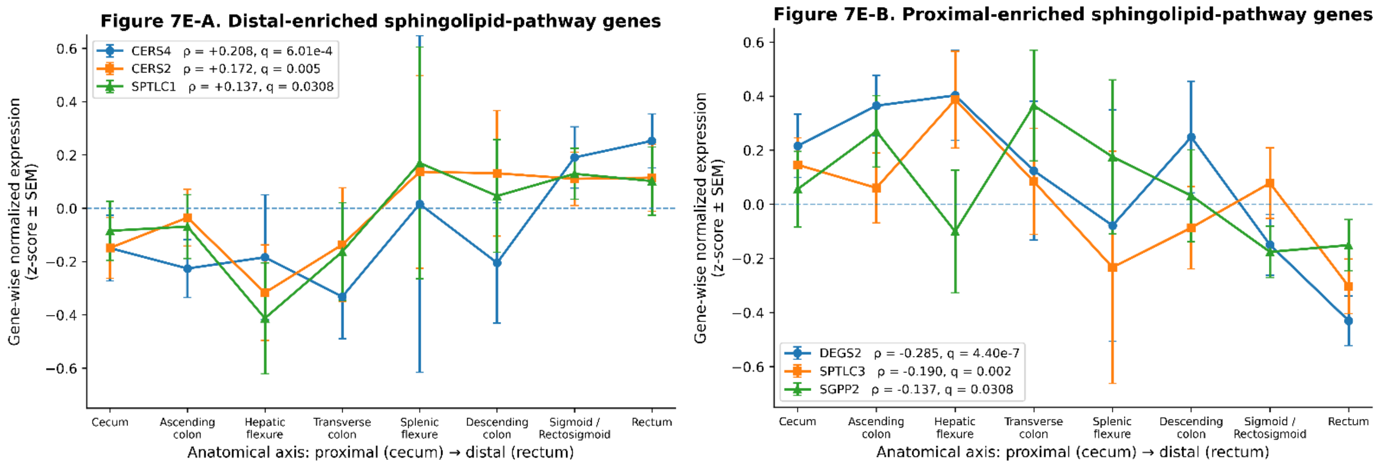

Supplementary Figure S1. Anatomical variation in sphingolipid-pathway gene expression along the cecum-to-rectum axis. Expression of the 23 evaluable sphingolipid-related genes was examined in 374 TCGA colorectal tumors with detailed anatomical annotation. Tumor locations were ordered along the cecum-to-rectum anatomical axis, and associations between gene expression and anatomical position were assessed using Spearman correlation with false discovery rate (FDR) correction. Six genes remained significantly associated with anatomical position after FDR correction: SPTLC1, CERS2, and CERS4 showed distal enrichment, whereas DEGS2, SPTLC3, and SGPP2 showed proximal enrichment. These findings represent gene-specific associations with ordered colorectal position rather than evidence of uniform expression gradients across consecutive anatomical segments.
